# Machine learning and response surface methodology for optimization and prediction of tribological performance of PLA/rice husk biochar composites

**DOI:** 10.1038/s41598-026-51463-5

**Published:** 2026-05-06

**Authors:** Sundarasetty Harishbabu, Amina Salhi, Leila Jamel, Borhen Louhichi, It Ee Lee, Santosh Kumar Sahu, Qamar Wali

**Affiliations:** 1 School of Mechanical Engineering, VIT-AP University, Besides A.P. Secretariat, Amaravati, 522237 Andhra Pradesh India; 2 Department of Information Systems, College of Computer and Information Sciences, Princess Nourah bint Abdulrahman University, Riyadh, 11671 Saudi Arabia; 3 Engineering Sciences Research Center (ESRC), Deanship of Scientific Research, Imam Mohammad Ibn Saud Islamic University (IMSIU), Riyadh, 11432 Saudi Arabia; 4 Faculty of Artificial Intelligence and Engineering, Multimedia University, Cyberjaya, 63100 Malaysia; 5 Centre for Smart Systems and Automation, COE for Robotics and Sensing Technologies, Multimedia University, Cyberjaya, 63100 Malaysia

**Keywords:** FDM, ANOVA, Machine learning, ANN, PLA, Biochar, Engineering, Materials science

## Abstract

The present study investigates the tribological behaviour of polylactic acid (PLA) composites reinforced with rice husk biochar (RHBC) through an integrated approach combining experimentation, statistical optimization, and machine learning. PLA/RHBC composites with filler contents ranging from 10 to 20 wt% were fabricated using fused deposition modeling (FDM). A Box–Behnken design (BBD) within the framework of Response Surface Methodology (RSM) was employed to systematically examine the effects of key process parameters—printing angle, infill type, nozzle temperature, and filler content at three levels and across 24 experimental runs. Tribological performance was evaluated in terms of wear rate and coefficient of friction (COF) using a pin-on-disc setup under applied loads of 10, 20, and 30 N. Analysis of variance (ANOVA) revealed that the applied load is the most significant factor influencing wear rate, whereas COF is predominantly governed by the combined effects of the printing parameters. Optical microscopy further indicated that incorporating RHBC reduces ploughing and improves interfacial stability. To enhance predictive capability, a machine learning framework incorporating Multiple Linear Regression (MLR), Extreme Gradient Boosting (XGBoost), and Artificial Neural Networks (ANN) was implemented. Among these, the ANN model demonstrated superior performance, achieving the highest prediction accuracy for wear rate with R² values of 0.9852, 0.9845, and 0.9891 at 10, 20, and 30 N, respectively. A similar trend was observed for COF, where ANN again outperformed the other models, yielding R² values of 0.9892, 0.9880, and 0.9910 at the corresponding loads. The integration of RSM with machine learning enables efficient optimization of FDM parameters and accurate prediction of tribological performance. This highlights the suitability of RHBC/PLA composites for lightweight, wear-resistant applications such as bearings, bushings, and sliding components.

## Introduction

As the world moves towards more sustainable practices, there’s a growing need to explore eco-friendly materials that can offer the same durability and performance as traditional ones^1^. Materials like bio-based plastics and biodegradable polymers such as polylactic acids (PLA) are at the forefront of this shift, offering alternatives that are both environmentally friendly and functional^2^. However, these materials often face challenges such as wear, degradation, and reduced strength in specific applications. By incorporating fillers, researchers can enhance the performance of these sustainable materials, improving their resistance to wear while maintaining their eco-friendly characteristics^3,4^. This approach could pave the way for creating more durable, sustainable alternatives to conventional materials, ultimately reducing environmental impact and promoting a greener future^5,6^.

Several studies have investigated the tribological performance of polylactic acid (PLA) composites, with a focus on improving wear resistance. Laraba et al.^7^ reported that PLA composites reinforced with graphene oxide (GO) exhibited nearly 50% lower wear rate than neat PLA under normal loads of 5 N and 8 N. Similarly, Al Abir et al.^8^ demonstrated that the incorporation of short carbon fibers and graphene nanoparticles in FFF-printed PLA significantly improved wear resistance, with the optimized formulation (≈ 5 wt% SCF + 5 wt% graphene) achieving nearly a fivefold reduction in specific wear rate compared to unreinforced PLA. Upadhyay et al.^9^ evaluated FDM-printed PLA exposed to simulated seawater for 30 days and observed controlled wear rates (3.3–6.5 × 10⁻⁶ mm³/N·m) despite surface pitting caused by salt deposition. Natural filler-based PLA composites have also shown promising wear performance. Fouly et al.^10^ developed a PLA–corn cob composite and reported a ~ 150% improvement in wear resistance with 20 wt% filler. Likewise, Hanon et al.^11^ found that the addition of bronze particles to 3D-printed PLA significantly reduced wear, while printing orientation strongly influenced tribological behavior. Processing parameters and reinforcement materials also play a key role in wear performance. Zawadzki et al.^12^ showed that carbon fiber reinforcement and optimized extrusion temperature improved the wear resistance of PLA, while simpler internal structures minimized material loss during sliding. Santo et al.^13^ reported that the addition of only 0.5 wt% graphene significantly reduced friction and improved wear behavior by modifying the dominant wear mechanism. Similarly, Keshavamurthy et al.^14^ observed that incorporating 5–10 wt% boron nitride enhanced the wear resistance of FDM-printed PLA parts. Bio-based fillers have also been explored to improve wear performance. Albahkali et al.^15^ showed that PLA reinforced with 10 wt% date-pit filler exhibited improved wear resistance after heat treatment, and predictive models successfully estimated wear volume. In another study, Lasch et al.^16^ compared neat PLA and bronze-filled PLA, reporting that bronze reinforcement drastically reduced wear rate from 16.7 × 10⁻⁴ to ~ 0.9 × 10⁻⁵ mm³/N·m. Other studies focused on tribological properties of PLA filled with biochar. Arunadevi et al.^17^ developed K-nearest neighbour (KNN) and ANN models to predict the tribological and mechanical properties of FDM-printed carbon fibre-reinforced PLA by varying infill density, orientation, layer height, and printing speed. Their best results were obtained with KNN (RMSE = 2.55, R² = 0.994), while the best ANN model gave RMSE = 3.72 and R² = 0.984, indicating strong prediction accuracy. Raj et al.^18^ adopted citrus maxima fruit peel biochar as reinforcement in PLA to investigate wear behaviour. The composite sample with 2 vol% biochar and 50% infill density showed reduced wear loss and COF.

Considerable research has been conducted to enhance the tribological performance of PLA composites by incorporating various fillers. Among these, rice husk biochar (RHBC) reinforcement is attracting significant attention due to its low cost, renewable origin, and porous carbon-rich structure^19,20^. However, the tribological behaviour of RHBC-reinforced PLA composites fabricated through additive manufacturing remains underexplored. Additive manufacturing, particularly fused deposition modeling (FDM) or 3D printing, provides greater control over processing parameters and internal material architecture, which can significantly influence tribological performance^21^. While most existing studies primarily rely on conventional statistical methods to analyse processing parameters, these approaches often struggle to capture the complex interactions among parameters and tribological responses. In recent years, machine learning has emerged as a powerful tool for modelling complex relationships and improving prediction accuracy in materials research^22^. Therefore, the novelty of this study lies in developing a hybrid approach that integrates experimental and statistical methods with advanced machine learning frameworks to optimize and predict the tribological behaviour **of** PLA composites reinforced with RHBC. The objective of this study is to fabricate 3D-printed RHBC/PLA composites and develop machine learning-based predictive models to analyze their tribological performance.

## Materials and methods

### Material

**Fig. 1:**
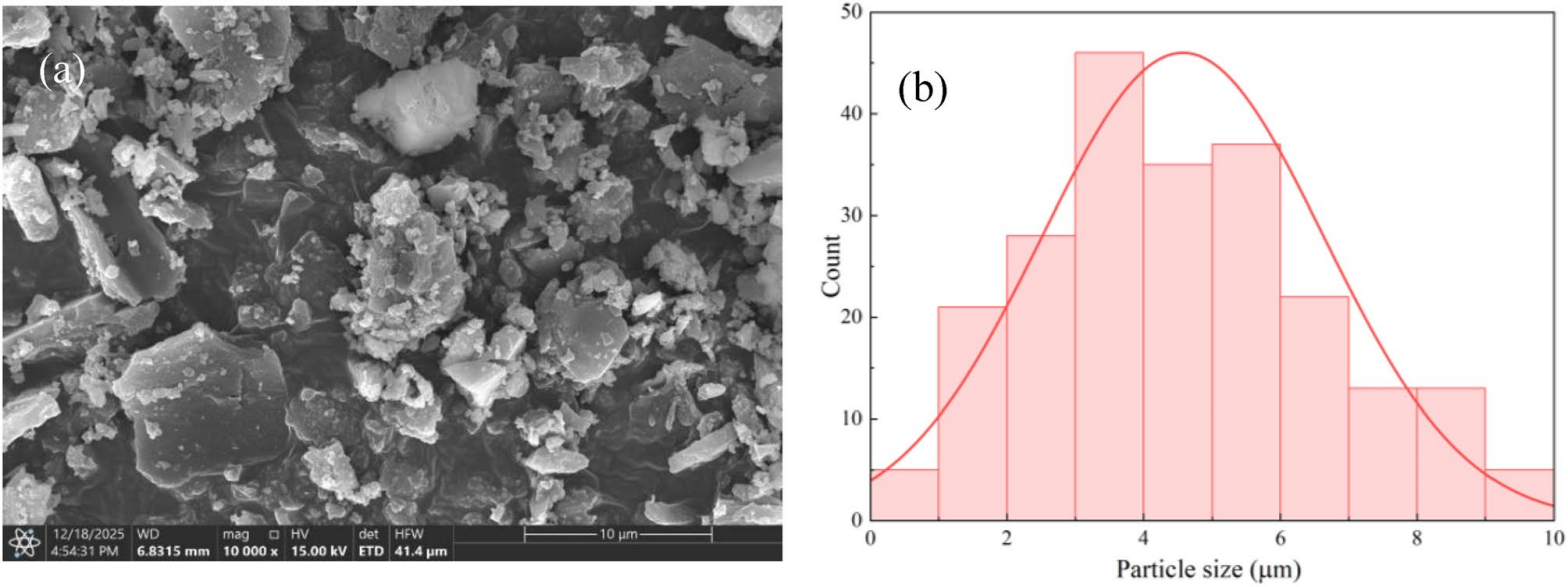
(**a**) SEM image of RHBC; (**b**) RHBC particle size distribution.

Polylactic acid (PLA) granules, with a specific gravity ranging from 1.30 to 1.35 g/cc, a melt flow rate of 2–4 g/10 cc, and a tensile strength of 25–35 MPa, were supplied by Deltora Biopolymer Pvt. Ltd., Ahmedabad, Gujarat, India. Rice husk biochar (RHBC), characterized by a fragmented and uneven surface with irregular shapes, as observed by scanning electron microscopy (SEM) and all particle sizes of less than 10 μm, a mean particle size of 4–5 μm was supplied by Vistarah Innovation Pvt. Ltd., Amaravathi, Andhra Pradesh, India. The microstructural analysis of supplied RHBC is shown in Fig. 1a, which characterized by fragmented particle of irregular structures. The particle size was analysed through image J software (v1.54), and was confirmed that the average particle size was less than 5 μm.

### Data analysis and experimental design

#### Experimental planning and factorial design

The experimental plan was developed using a combined statistical and data-driven modeling framework, which was employed to systematically analyze the responses obtained from the experimental analysis and also establish robust relationships between printing parameters, including reinforcement, and tribological properties. The Design-Expert^®^ (version 13) was employed to plan the experiments within the framework of a Box-Behnken design (BBD) of response surface methodology (RSM) for the evaluation of linear and nonlinear effects of key parameters, including filler wt%, printing orientation angle, infill type, and nozzle temperature, each considered at three levels as shown in Table 1. To ensure statistical reliability, a total of 24 randomized experimental runs were generated, as shown in Table 2. Conventional statistical tools were initially used to assess factor significance and interaction effects, followed by advanced data-driven modeling techniques to improve the predictive accuracy of tribological properties of RHBC/PLA composites^23,24^.

**Table 1:** Control factors and their corresponding levels for experimental design.

Levels Factor	Filler wt% (*P*_1_)	Printing angle (^o^) (*P*_2_)	Infill type (*P*_3_)	Nozzle temperature(°C) (*P*_4_)
Level 1	0 (pure PLA)	0	Hexagon	190
Level 2	10	60	Triangle	200
Level 3	20	120	3D infill	210

**Table 2:** Box-Behnken design (BBD) experimental matrix.

Run	Filler wt%	Printing angle (^o^)	Infill type	Nozzle temperature (°C)
R1	10	0	Triangle	210
R2	0	0	Triangle	200
R3	10	60	3D infill	190
R4	10	60	3D infill	210
R5	10	0	3D infill	200
R6	0	60	Triangle	190
R7	10	120	Triangle	190
R8	10	120	Hexagon	200
R9	0	60	Hexagon	200
R10	10	120	Triangle	210
R11	20	60	Triangle	210
R12	0	120	Triangle	200
R13	0	60	Triangle	210
R14	20	60	3D infill	200
R15	20	60	Triangle	190
R16	20	60	Hexagon	200
R17	10	60	Hexagon	190
R18	10	0	Hexagon	200
R19	20	120	Triangle	200
R20	10	0	Triangle	190
R21	10	120	3D infill	200
R22	10	60	Hexagon	210
R23	0	60	3D infill	200
R24	20	0	Triangle	200

#### Analysis of Factor Contribution

The influence of printing parameters and filler wt% on the tribological behavior of PLA/RHBC composites was analyzed using analysis of variance (ANOVA), which was performed to evaluate the influence of individual factors and their interactions. To capture the linear interaction and second-order effects, a quadratic regression model was employed. The contribution for each factor is identified based on F-values and corresponding p-values at a 95% confidence level. This analysis provided insight into dominant parameters governing material behavior and served as a baseline for comparison^25,26^.

### Processing and sample fabrication

Samples were processed using fused deposition modeling (FDM) on a 3D printer, in accordance with ASTM standards, to analyze the tribological behavior. The process included composition formulation, filament extrusion, and sample printing.

#### Composition formation and filament extrusion

**Fig. 2:**
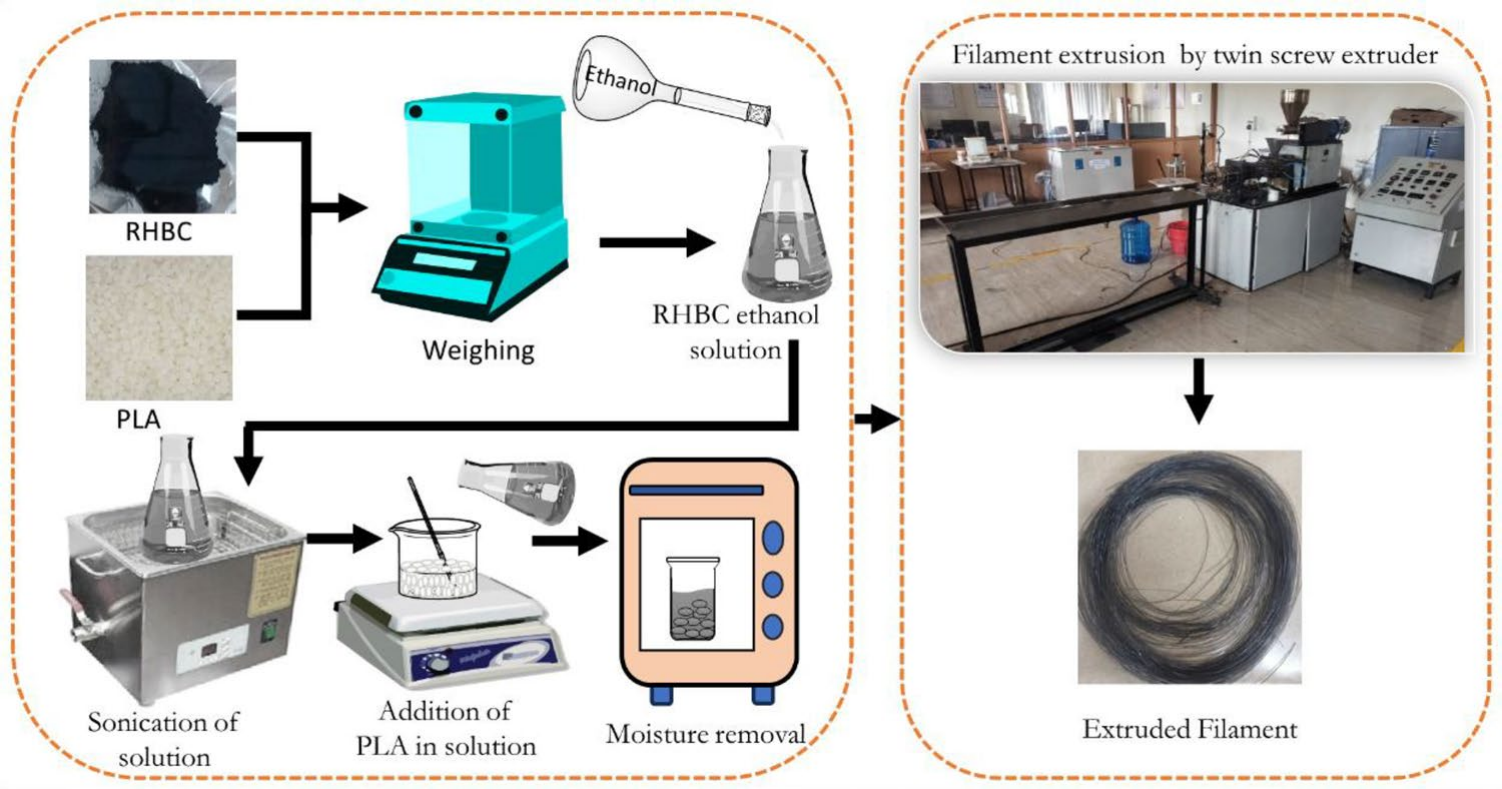
Steps for the filament extrusion process.

The filament extrusion process involves converting granular PLA and Rice husk biochar (RHBC) into filament, as shown in Fig. 2. In this process, the PLA was mixed with RHBC at 10% and 20%, measured using a digital balance, then mixed with ethanol in a 20:1 ratio. The biofiller mixture is sonicated for a period of 30 min at a frequency of 40 kHz to ensure uniform dispersion. The PLA granules were added to the sonicated fluid on a hot plate and continuously stirred. The mixture is then dried in a vacuum oven at 70 °C for 24 h to remove moisture. The dried RHBC/PLA composite granules are used for filament extrusion. A twin-screw extruder (AASAVI/25TS/CO/300/30, Aasabi Machinery Pvt. Ltd., Mumbai, India) is used to extrude the filament, with barrel temperatures ranging from 170 °C to 210 °C and a 2 mm die diameter at 30 rpm. The molten composite is rapidly cooled in a water bath to produce a filament with a diameter of 1.75 ± 0.05 mm. The filament is then stored in an airtight container to prevent moisture absorption^27,28^.

#### Sample printing

**Fig. 3:**
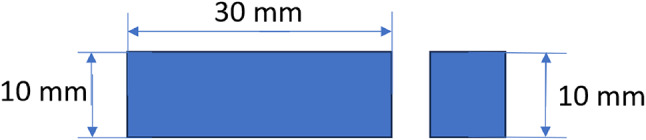
Tribology test sample.

The sample geometries are designed according to ASTM G99, as shown in Fig. 3. The FlashForge FDM 3D printer with a 0.4 mm nozzle is used to print the samples, with parameters adjusted according to the experimental design in Table 2. The samples are printed with a wall thickness of 1.6 mm and 100% infill density to ensure solid internal structures. The printed samples are inspected for defects and dimensional accuracy in accordance. After printing, the samples are prepared for tribology testing^29^.

### Tribological test

The tribological characterisation of RHBC/PLA composites was carried out using a pin-on-disc testing machine (DOCOM TR-20LE-PHM400-CHM400) under ambient conditions in accordance with ASTM G99. The variable printing parameters, including filler wt%, were analysed using experimental designs based on response surface methods (RSM), as shown in Table 2, at a sliding speed and sliding distance of 300 RPM and 1500 mm, respectively, by varying the applied load to 10 N, 20 N, and 30 N. The wear rate and the coefficient of friction (COF) were estimated from the experimental results by using Eqs. (1–3)^30^ for each experimental condition, which was repeated three times to ensure accuracy, and the average values were reported for analysis^31,32^.

1 $$W\:\frac{\mathrm{V}}{{F.\:D}}$$

2 $$V=d\cdot{A}$$

3 $$\mu=\:\frac{{\mathrm{F}}_{f}}{\mathrm{F}}$$

Where, W= wear rate (µm^3^/(N.m)), V = worn volume (m³), F = applied normal load (N), D = sliding distance (m), d = wear depth (m), A = contact area of the specimen (m²), F_*f*_​ = frictional force (N), and µ = coefficient of friction.

### Optical microscopy

The wear characteristics of the tested samples, the morphology of the worn surfaces, and wear tracks after tribological testing were examined under Optical microscopy. Following the wear experiments, the samples were gently cleaned with ethanol to remove loose debris and dried under ambient conditions. The worn regions were then observed using an optical microscope equipped with a digital camera, operating in reflected light mode at different magnifications.

### Machine learning

The machine learning framework was adopted to predict the tribological behavior of RHBC/PLA composites. Three regression algorithms, namely multiple linear regression (MLR), extreme gradient boosting (XGBoost), and artificial neural networks (ANN), were implemented to provide a comparative evaluation of linear, tree-based, and nonlinear predictive approaches. MLR was selected as a baseline model due to its simplicity and interpretability, whereas XGBoost was chosen for its robustness and ability to capture nonlinear feature interactions with strong resistance to overfitting. ANN was employed for its ability to learn complex nonlinear relationships among multiple process parameters and tribological responses. To obtain the best predictive performance, the models were optimized using Grid Search Cross-Validation (GridSearchCV). Model performance was evaluated using statistical metrics such as the coefficient of determination (R²), root mean square error (RMSE), and mean absolute error (MAE), as given in Eqs. (4)-(6)^33,34^. In addition, SHAP-based explainability was employed to interpret feature importance and provide physical insights into the learned relationships between input parameters and output responses.

4 $$\:{\mathrm{R}}^{2}=1-\frac{\sum\:_{\mathrm{a}=1}^{b}{{(\mathrm{k}}_{\mathrm{a}}-{\widehat{\mathrm{k}}}_{\mathrm{a}})}^{2}}{\sum\:_{\mathrm{a}=1}^{b}{{(\mathrm{k}}_{\mathrm{a}}-\stackrel{-}{\mathrm{k}})}^{2}}$$

5 $$\:\mathrm{R}\mathrm{M}\mathrm{S}\mathrm{E}=\sqrt{\mathrm{M}\mathrm{S}\mathrm{E}}=\:\sqrt{\frac{1}{\mathrm{b}}\sum\:_{\mathrm{a}=1}^{\mathrm{l}}{{(\mathrm{k}}_{\mathrm{a}}-{\widehat{\mathrm{k}}}_{\mathrm{a}})}^{2}}$$

6 $${\mathrm{MAE}} = \:\frac{1}{{\mathrm{b}}}\sum {\:_{{{\mathrm{a}} = 1}}^{{\mathrm{b}}} } \left| {({\mathrm{k}}_{{\mathrm{a}}} - {{\hat{k}}}_{{\mathrm{a}}} } \right|\:$$

Here, *b* is the total number of observations or trials, k_a_ is actual measured value for a^th^ trial, while $\:{\widehat{\mathrm{k}}}_{\mathrm{a}}$ is the predicted value of the response variable for the i^th^ observation. $\:\stackrel{-}{\mathrm{k}}$ represents mean of all observed values.

#### MLR

To predict the tribological behaviour of RHBC/PLA composites with multiple targets, such as wear rate and coefficient of friction, under varying input parameters, multiple linear regression (MLR) was employed. In a general linear regression model, the model was trained separately for each target to predict. This approach enables us to predict multiple outcomes based on variable input parameters. For the model evaluation, the data sets were split into training and testing sets. Equation (7) presents the relation between the input parameter and the target variables^35^.

7 $$\:\widehat{k}={\mathrm{b}}_{0}+{\mathrm{b}}_{1}\mathrm{A}+{\mathrm{b}}_{2}\mathrm{B}+{\mathrm{b}}_{3}\mathrm{C}+{\mathrm{b}}_{4}\mathrm{D}$$

Here, $\:\widehat{k}$ is the predicted response variable, and A, B, C, and D are independent variables, $\:{\mathrm{b}}_{0}$ is the intercept of the equation, and $\:{\mathrm{b}}_{1},\:{\mathrm{b}}_{2},\:{\mathrm{b}}_{3}$, and $\:{\mathrm{b}}_{4}$ are regression coefficients.

#### XG boosting

Extreme Gradient Boosting (XGBoost) is the most commonly used tree-based ensemble learning approach for predicting model accuracy, due to its numerical stability and strong resistance to overfitting. The algorithm differs from conventional gradient boosting techniques by explicitly penalising model complexity through the inclusion of both L1 and L2 regularisation terms in the optimisation objective. This formulation improves predictive reliability by balancing data fitting with structural simplicity. In the present study, XGBoost is implemented to model the tribological behaviour of RHBC/PLA composites, focusing on the wear rate and the coefficient of friction. The input variables comprise the key 3D printing parameters, namely orientation angle, infill type, nozzle temperature, and filler wt%. Model training is carried out by iteratively constructing regression trees that minimise a regularised objective function, as expressed in Eqs. (8) and (9)^36,37^, where the loss associated with prediction errors is jointly optimised with penalties related to tree complexity and leaf weight magnitude.

8 $$\user2{L}^{{\left( \user2{t} \right)}} = \mathop \sum \limits_{{\user2{i} = 1}}^{\user2{n}} \user2{l}\left( {\user2{k}_{\user2{i}} ,\user2{~\hat{k}}_{\user2{i}} ^{{\left( \user2{t} \right)}} } \right) + \mathop \sum \limits_{{\user2{k} = 1}}^{\user2{t}} \emptyset \left( {\user2{f}_{\user2{k}} } \right)$$

9 $$\emptyset \:\left( {f_{k} } \right) = \gamma \:T + \frac{1}{2}\lambda \:\left\| \omega \right\|^{2}$$

Here $\:\boldsymbol{l}$ is a differentiable loss function, $\:{\boldsymbol{k}}_{\boldsymbol{i}}$ and $\:{\widehat{\boldsymbol{k}}}_{\boldsymbol{i}}$ are actual and predicted values for the i^th^ data point, $\:\boldsymbol{\varnothing\:}\left({\boldsymbol{f}}_{\boldsymbol{k}}\right)$ represents a regularisation term controlling model complexity at iteration 𝒌, 𝑻 is the number of leaves in the tree, 𝝎 is the vector of leaf weights, and 𝜸, 𝝀 are regularisation parameters.

#### ANN

Artificial Neural Network (ANN) was employed to estimate multiple target variables from the input parameters of the 3D printer based on a Multi-Layer Perceptron (MLP). The MLP is a fully connected feed-forward network that contains an input layer, one hidden layer, and an output layer, enabling it to learn nonlinear relationships between variables. In this work, the ANN was implemented using scikit-learn’s MLP Regressor with one hidden layer of 50 neurons, a maximum of 800 training iterations, and L2 regularization controlled by $\:{\upalpha\:}=0.001$. Because the response consists of multiple output variables, a separate MLP model was trained for each output dimension, and the final prediction vector was obtained by stacking the individual model predictions. The relation between input and target parameters presented in equations (10) to (11)^38^.

10 $$\:{\mathrm{z}}_{j}=\sum\:_{i=1}^{n}{\mathrm{W}}_{1,ji}{\mathrm{x}}_{i}+{\mathrm{b}}_{1,j}$$

11 $$\:{\widehat{k}}_{r}\:=\:\sum\:_{\mathrm{j}=1}^{m}{\mathrm{W}}_{2,rj}{\beta\:(\mathrm{z}}_{j})+{\mathrm{b}}_{2,\mathrm{r}}$$

Here, x is *i*^th^ input features, *n* is number of input features, m is number of hidden neurons, $\:{\mathrm{W}}_{1,ji}$ is weight connecting to input *i* to hidden neuron *j*, $\:{\mathrm{b}}_{1,j}$ is the bias of *j*^th^ hidden neuron, $\:{\mathrm{z}}_{j}$ is the pre-activation output of hidden neuron *j*, $\:{\beta\:(\mathrm{z}}_{j})$ is the ReLU activation function defined as max (0, $\:{\mathrm{z}}_{j}$), $\:{\mathrm{W}}_{2,rj}$ is the weight connecting hidden neuron *j* to output neuron r, b_2,r_ is bias of the r^th^ output neurons, $\:{\widehat{k}}_{r}\:$is the predicted output.

## Results and discussion

### Experimental wear results

**Fig. 4:**
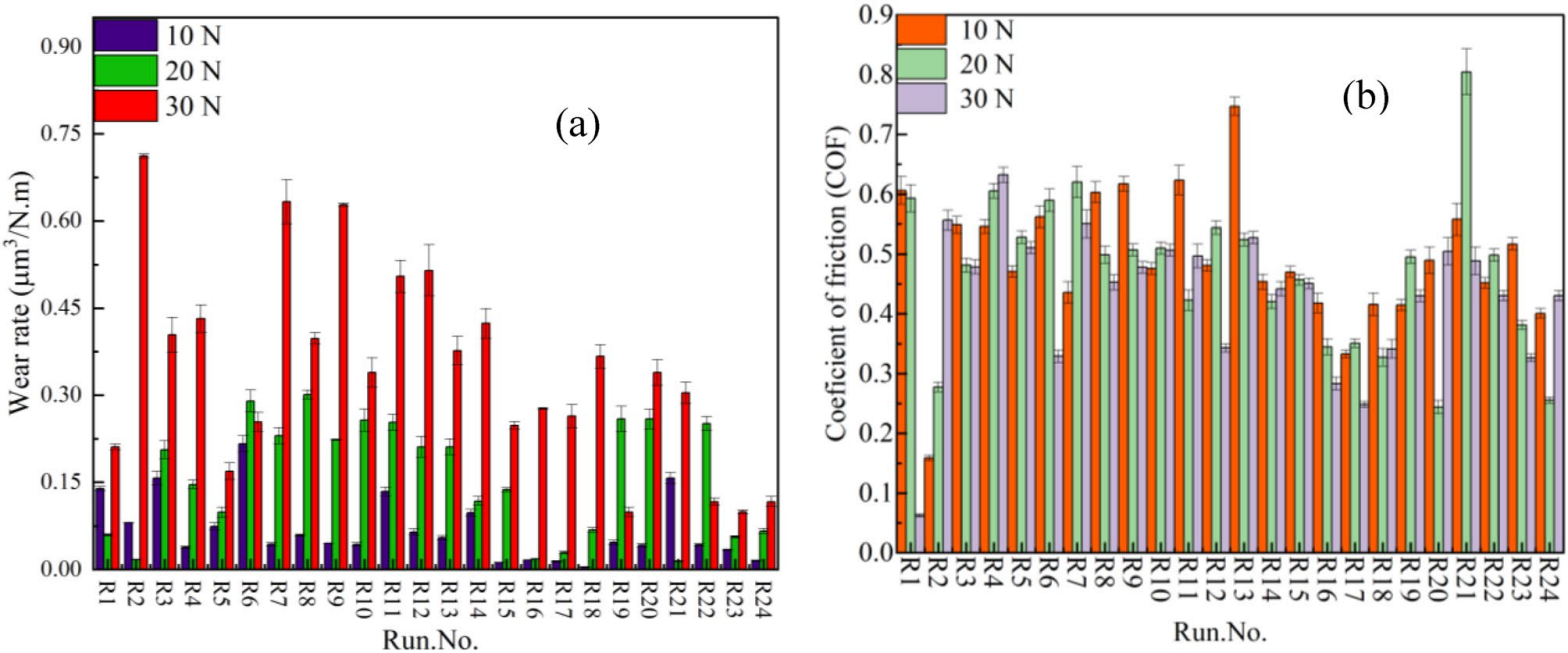
Testing results (**a**) Wear rate and (**b**) coefficient of friction (COF).

Figure 4(a-b) presents the experimentally obtained tribological results for 24 samples designed using statistical analysis, varying printing parameters, including filler wt%, under three different loads of 10 N, 20 N, and 30 N, as shown in the Table 3. Figure 4(a) presents a progressive increase in wear rate with increasing load. At 10 N, the lowest wear is observed in R18 (0.00438 μm³/N.m), followed by R17 (0.0143 μm³/N.m) and R16 (0.0162 μm³/N.m), while the highest wear occurs in R6 (0.216 μm³/N.m). At 20 N, R21 (0.0145 μm³/N.m) and R2 (0.0172 μm³/N.m) exhibit minimal wear, whereas R8 (0.301 μm³/N.m) and R6 (0.290 μm³/N.m) show greater material removal. Under 30 N, wear increases sharply, reaching a maximum at R2 (0.712 μm³/N · m), followed by R7 (0.633 μm³/N · m) and R9 (0.628 μm³/N · m). Figure 4(b) demonstrates the corresponding COF values, which vary nonlinearly with load; at 10 N, COF ranges from 0.159 (R2) to 0.747 (R13), at 20 N from 0.244 (R20) to 0.8045 (R21), and at 30 N from 0.248 (R17) to 0.632 (R4). The differing trends confirm that wear is primarily load-driven, whereas friction depends on interfacial adhesion and transfer film behavior.

**Table 3:** Experimental responses for the factorial design.

Run	Wear rate at 10 *N* (µm^3^/Nm)	Wear rate at 20 *N* (µm^3^/Nm)	Wear rate at 30 *N* (µm^3^/Nm)	COF at 10 *N*	COF at 20 *N*	COF at 30 *N*
R1	0.139	0.0599	0.211	0.606	0.593	0.0623333
R2	0.0805	0.0172	0.712	0.159	0.2775	0.556333
R3	0.157	0.206	0.404	0.549	0.4815	0.478667
R4	0.0383	0.146	0.432	0.546	0.6055	0.632333
R5	0.0743	0.099	0.169	0.471	0.5285	0.510667
R6	0.216	0.29	0.254	0.562	0.5895	0.329333
R7	0.0437	0.23	0.633	0.436	0.62	0.550333
R8	0.0593	0.301	0.398	0.603	0.499	0.453
R9	0.0453	0.223	0.628	0.617	0.507	0.478
R10	0.043	0.257	0.339	0.476	0.51	0.506667
R11	0.134	0.253	0.505	0.623	0.423	0.497
R12	0.0646	0.211	0.515	0.481	0.544	0.343
R13	0.0549	0.211	0.377	0.747	0.5245	0.527667
R14	0.0979	0.118	0.424	0.454	0.4205	0.442
R15	0.0121	0.137	0.248	0.47	0.457	0.451
R16	0.0162	0.0185	0.277	0.418	0.345	0.283333
R17	0.0143	0.0293	0.264	0.333	0.351	0.248333
R18	0.00438	0.0686	0.367	0.416	0.3275	0.341333
R19	0.0467	0.259	0.0992	0.415	0.4955	0.430333
R20	0.0418	0.259	0.339	0.49	0.244	0.504667
R21	0.157	0.0145	0.304	0.558	0.8045	0.488667
R22	0.0428	0.251	0.117	0.452	0.4985	0.430667
R23	0.034	0.0564	0.0995	0.517	0.3815	0.326667
R24	0.0154	0.0662	0.117	0.401	0.2555	0.430667

**Fig. 5:**
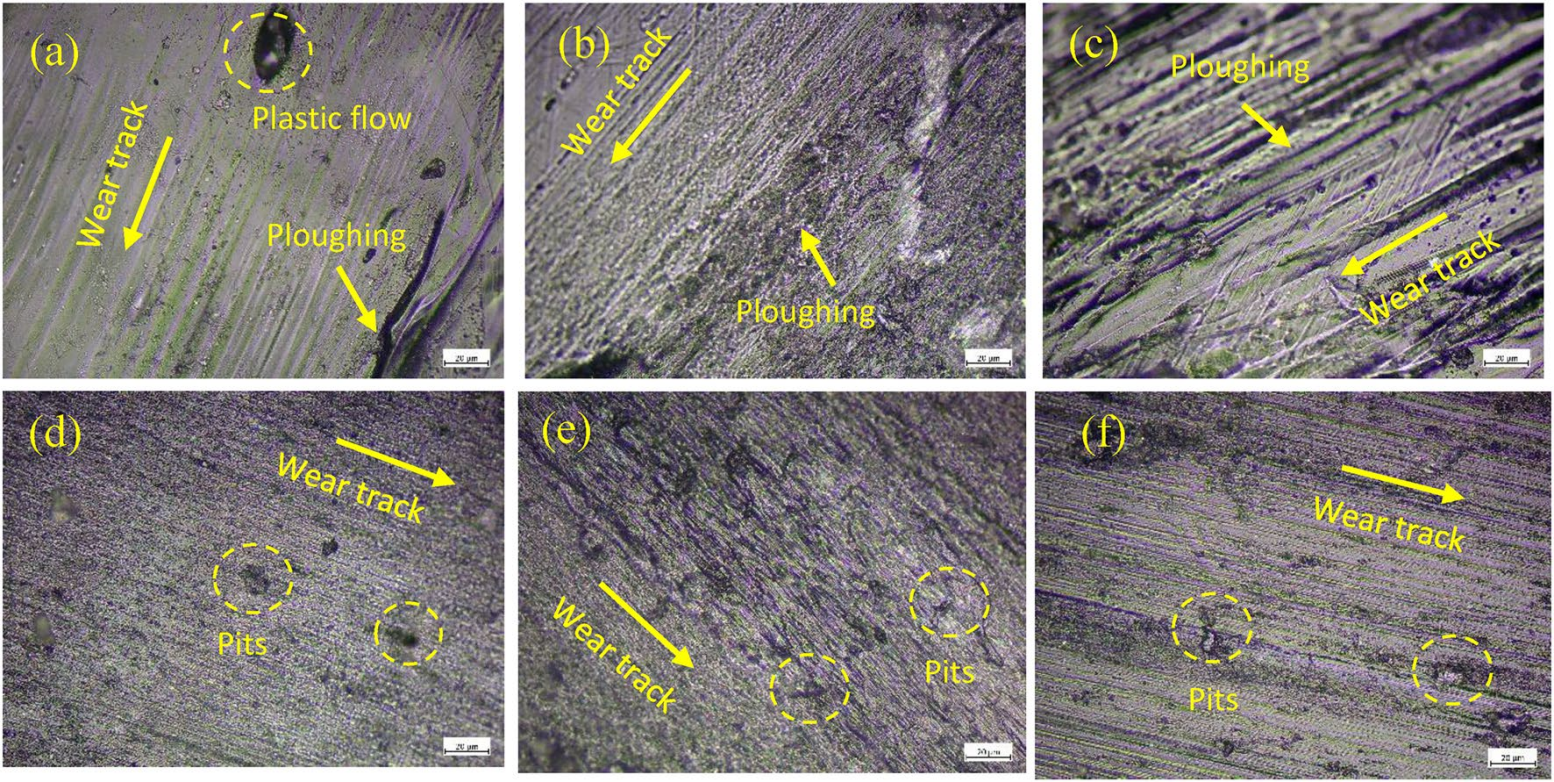
Wear track for pure PLA at (**a**) 10 N, (**b**) 20 N, (**c**) 30 N loads; and RHBC/PLA composites at (**d**) 10 N, (**e**) 20 N, (**f**) 30 N loads.

The optical images of the wear tracks reveal that the wear mechanism varies with both applied load and the presence of RHBC reinforcement. For pure PLA at 10 N load as shown in Fig. 5a, the worn surface predominantly shows plastic flow with relatively less ploughing, indicating that the polymer undergoes viscoelastic deformation under mild contact stress. As the load increases to 20 and 30 N as shown in Fig. 5b and c, it pronounced severe ploughing and groove formation, indicating a transition from mild deformation to severe abrasive wear, accompanied by increased material removal and surface damage. For the 20 wt% PLA/RHBC composite, the wear mechanism differs due to the presence of rigid rice husk biochar (RHBC) particles dispersed within the PLA matrix. At 10 N load as shown in Fig. 5d, the worn surface exhibits pits, which are mainly attributed to the pull-out of RHBC particles from the PLA matrix during sliding. As the load increases to 20 and 30 N as shown in Fig. 5e and f, the interfacial stress between the matrix and reinforcement becomes higher, resulting in more frequent particle debonding and pull-out, which leads to a greater number of pits on the worn surface. Additionally, the detached RHBC particles may act as third-body abrasives, contributing to localized surface damage while forming a carbonaceous layer that influences the overall wear behavior of the composite.

### Statistical data analysis

#### Wear rate

Table 4 illustrates the analysis of factor contribution for wear rate under different loading conditions, such as 10 N, 20 N, and 30 N. At a load of 10 N, the model yields F = 21.83, with *p* < 0.05, indicating a highly significant result, with R^2^ = 0.9762 and Adj. R^2^ =0.9314, indicating an excellent fit, with most influential factors being infill type (P_3_), followed by filler content (P_1_), printing angle (P_2_), and nozzle temperature (P_4_), while strong interactions such as with F and p-values P_1_×P_4_ and P_3_×P_4_ with p-value less than 0.05. Similarly, for the 20 N load, the model remains statistically significant, with F values of 6.61 and p-values less than 0.05, indicating a best fit with R^2^ values of 0.9140 and 0.9433, and Adj. R^2^ of 0.7526; printing angle (P_2_) and interaction terms like P_2_×P_3_ and (P_3_²) dominate, while P_1_ becomes statistically insignificant with a p-value greater than 0.05. At 30 N, the model again shows F = 8.88, *p* = 0.0020, and strong significance, with R² = 0.9433 and Adj. R² = 0.8371, where P2, P_3_, and P_4_ significantly affect wear, along with high-order interactions such as P_1_×P_2_² and P_3_×P_4_², indicating that at higher loads, wear is governed mainly by orientation, infill architecture, and temperature interactions rather than filler content alone for variation of loads.

**Table 4:** Analysis of factor contribution using ANOVA for wear rate.

Load: 10 *N*				Load: 20 *N*			Load: 30 *N*			df
Source	F-value	p-value		Source	F-value	p-value	Source	F-value	p-value	
Model	21.83	< 0.0001		Model	6.61	0.0055	Model	8.88	0.0020	15
P_1_	26.04	0.0009		P_1_	3.90	0.0838	P_1_	0.2374	0.6391	1
P_2_	10.68	0.0114		P_2_	6.04	0.0395	P_2_	9.00	0.0171	1
P_3_	56.39	< 0.0001		P_3_	0.0013	0.9723	P_3_	11.81	0.0089	1
P_4_	10.10	0.0130		P_4_	2.39	0.1609	P_4_	7.62	0.0247	1
P_1_$\:\times\:$P_3_	10.43	0.0121		P_1_$\:\times\:$P_3_	8.56	0.0191	P_1_$\:\times\:$P_2_	1.67	0.2321	1
P_1_$\:\times\:$P_4_	96.58	< 0.0001		P_2_$\:\times\:$P_3_	12.15	0.0083	P_1_$\:\times\:$P_3_	23.76	0.0012	1
P_2_$\:\times\:$P_4_	11.56	0.0094		P_2_$\:\times\:$P_4_	6.18	0.0377	P_1_$\:\times\:$P_4_	0.9349	0.3619	1
P_3_$\:\times\:$P_4_	26.13	0.0009		P_3_$\:\times\:$P_4_	9.60	0.0147	P_2_$\:\times\:$P_3_	0.5632	0.4745	1
$\:{\mathrm{P}}_{1}^{2}$	7.36	0.0265		$\:{\mathrm{P}}_{1}^{2}$	6.65	0.0327	P_2_$\:\times\:$P_4_	1.43	0.2653	1
$\:{\mathrm{P}}_{1}^{2}\times\:{\mathrm{P}}_{2}$	7.23	0.0275		$\:{\mathrm{P}}_{2\:}^{2}$	7.17	0.0281	P_3_$\:\times\:$P_4_	1.59	0.2422	1
$\:{\mathrm{P}}_{1}^{2}\times\:{\mathrm{P}}_{3}$	5.47	0.0475		$\:{\mathrm{P}}_{3\:}^{2}$	19.25	0.0023	$\:{\mathrm{P}}_{1}^{2}$	0.6741	0.4354	1
$\:{\mathrm{P}}_{1}\times\:{\mathrm{P}}_{3}^{2}$	15.47	0.0043		$\:{\mathrm{P}}_{1}^{2}\times\:{\mathrm{P}}_{2}$	4.21	0.0742	$\:{\mathrm{P}}_{1}^{2}\times\:{\mathrm{P}}_{2}$	8.99	0.0171	1
$\:{\mathrm{P}}_{2}^{2}\times\:{\mathrm{P}}_{4}$	20.89	0.0018		$\:{\mathrm{P}}_{1}\times\:{\mathrm{P}}_{2}^{2}$	4.04	0.0792	$\:{\mathrm{P}}_{1}^{2}\times\:{\mathrm{P}}_{4}$	14.69	0.0050	1
$\:{\mathrm{P}}_{2}\times\:{\mathrm{P}}_{3}^{2}$	32.38	0.0005		$\:{\mathrm{P}}_{2}^{2}\times\:{\mathrm{P}}_{3}$	5.38	0.0489	$\:{\mathrm{P}}_{1}\times\:{\mathrm{P}}_{2}^{2}$	38.90	0.0002	1
$\:{\mathrm{P}}_{1}^{2}\times\:{\mathrm{P}}_{4}^{2}$	37.75	0.0003		$\:{\mathrm{P}}_{2}^{2}\times\:{\mathrm{P}}_{4}$	5.94	0.0407	$\:{\mathrm{P}}_{3}\times\:{\mathrm{P}}_{4}^{2}$	21.76	0.0016	1
Residual										8
Cor Total										23
R^2^ = 0.9762, Adj. R^2^= 0.9314			R^2^ = 0.9140, Adj. R^2^= 0.7526				R^2^ = 0.9433, Adj. R^2^= 0.8371			

**Fig. 6:**
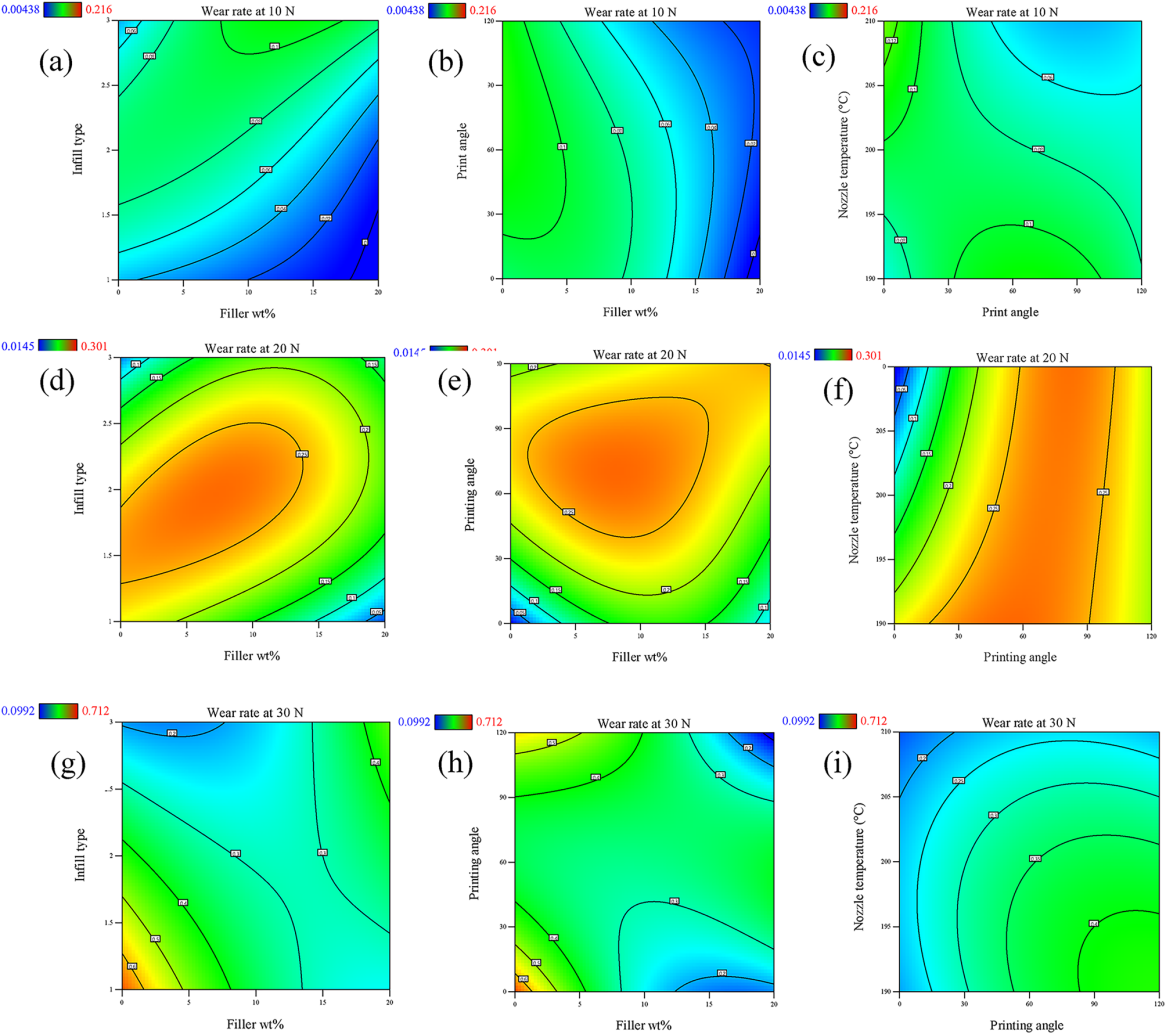
Response surface contour plots showing parameter interactions for wear rate at 10 N: (**a**) filler wt%–infill type, (**b**) filler wt%–printing angle, (**c**) printing angle–nozzle temperature; at 20 N: (**d**) filler wt%–infill type, (**e**) filler wt%–printing angle, (**f**) printing angle–nozzle temperature; and at 30 N: (**g**) filler wt%–infill type, (**h**) filler wt%–printing angle, (**i**) printing angle–nozzle temperature.

The combined effects of printing parameters on the wear rate of RHBC/PLA composites under varying normal loads using counter plots are shown in Fig. 6(a) to 6(i). Figure 6(a–c) corresponds to a load of 10 N; the color contours are smoothly distributed and in the lower range, with the interaction between filler wt% and infill type, filler wt% and printing angle, and printing angle with nozzle temperature indicating mild wear dominated by adhesive mechanisms and stable sliding conditions. Figure 6(d–f) presents 20 N, exhibits a broader count and shift towards wider high-wear regions (orange–red zones). This reveals that intermediate filler content combined with unfavorable infill type or printing angle leads to increased wear due to higher contact stress and the onset of abrasive action. Figure 6(g-i) demonstrates that at 30 N, the contour lines become steeper and uneven, representing the strong sensitivity of wear rate to parameter interactions. A small change in filler wt% or nozzle temperature can significantly increase or reduce wear. Finally, the contour plots reveal that wear varies significantly with load, but optimised combinations of filler wt% and printing parameters significantly reduce wear.

#### Coefficient of friction

Table 5 demonstrates the factor contribution analysis for the coefficient of friction using ANOVA under varying loads of 10 N, 20 N and 30 N, highlighting that the developed model and the factor contributions, such as filler wt% (P_1_), printing angle (°) (P₂), infill type (P₃), and nozzle temperature (°C) (P₄) are statistically significant. The models are significant with a p-value less than 0.05, and F-values 11.11, 14.02 and 13.6, respectively, for all loading conditions conforms the selected parameters adequately explain variations in frictional behavior. At 10 N, filler wt% (P_1_) and infill type (P_3_) significantly influence the frictional behavior with less than 0.05 p-values and an F value of 15.24 and 12.82, respectively, showing significant influence on frictional behavior, along with interaction and quadratic effects such as P_1_$\:\times\:$P_2_, $\:{\mathrm{P}}_{1}^{2}$,$\:{\:\mathrm{P}}_{1}^{2}\times\:{\mathrm{P}}_{2}$, $\:{\mathrm{P}}_{1}^{2}\times\:{\mathrm{P}}_{3}$, $\:{\mathrm{P}}_{1}\times\:{\mathrm{P}}_{2}^{2}$, P₂²×P₃, P₂×P₃², P₁²×P₂², P₁²×P₃². Similarly, at 20 N, the coefficient of friction is predominantly governed by the printing angle (P₂), nozzle temperature (P₄), filler weight% (P₁), and infill type (P₃), which exhibits the strongest influence with an F-value of 67.10, 16.87, 7.89, 7.30 with p-value less than 0.05, further highlight the importance of coupled process effects at this load are P₁×P₃, P_2_×P₄, P₁²×P_3_, P₁²×P_4_, P₁²×P_2_², and P₁²×P₃². At the highest load of 30 N, the influence of process parameters becomes more pronounced, with printing angle (P₂), infill type (P₃), and nozzle temperature (P₄) with p-value less than 0.05 and F = 35.36, 27.49, *p* = 0.0005, and 24.80, respectively, emerging as the dominant factors controlling the coefficient of friction. Significant interaction and quadratic effects, such as P₁×P₂, P₁×P₃, P₁×P₄, P₁²×P₂, and P₂×P₄², indicate strong nonlinear and load-dependent interactions, reflecting the increasing role of contact mechanics and material deformation under severe loading conditions.

**Table 5:** Analysis of Factor Contribution using ANOVA for COF.

Load: 10 *N*			Load: 20 *N*			Load: 30 *N*			df
Source	F-value	p-value	Source	F-value	p-value	Source	F-value	p-value	
Model	11.11	0.0005	Model	14.02	0.0002	Model	13.6	0.0002	14
P_1_	15.25	0.0036	P_1_	7.89	0.0204	P_1_	0.0349	0.8559	1
P_2_	4.52	0.0625	P_2_	67.1	< 0.0001	P_2_	35.36	0.0002	1
P_3_	12.82	0.0059	P_3_	7.3	0.0243	P_3_	27.49	0.0005	1
P_4_	4.94	0.0534	P_4_	16.87	0.0026	P_4_	24.8	0.0008	1
P_1_$\:\times\:$P_2_	12.66	0.0061	P_1_$\:\times\:$P_2_	0.0909	0.7699	P_1_$\:\times\:$P_2_	6.68	0.0295	1
$\:{\mathrm{P}}_{1}^{2}$	17.94	0.0022	P_1_$\:\times\:$P_3_	5.23	0.048	P_1_$\:\times\:$P_3_	14.15	0.0045	1
$\:{\mathrm{P}}_{1}^{2}\times\:{\mathrm{P}}_{2}$	18.04	0.0021	P_2_$\:\times\:$P_3_	1.41	0.2649	P_1_$\:\times\:$P_4_	3.42	0.0975	1
$\:{\mathrm{P}}_{1}^{2}\times\:{\mathrm{P}}_{3}$	9.33	0.0137	P_2_$\:\times\:$P_4_	27.27	0.0005	P_1_$\:\times\:$P_4_	23.41	0.0009	1
$\:{\mathrm{P}}_{1}^{2}\times\:{\mathrm{P}}_{4}$	3.63	0.0891	$\:{\mathrm{P}}_{1}^{2}$	0.0708	0.7962	$\:{\mathrm{P}}_{1}^{2}\times\:{\mathrm{P}}_{2}$	36.46	0.0002	1
$\:{\mathrm{P}}_{1}\times\:{\mathrm{P}}_{2}^{2}$	15.32	0.0035	$\:{\mathrm{P}}_{1}^{2}\times\:{\mathrm{P}}_{3}$	5.35	0.046	$\:{\mathrm{P}}_{1}^{2}\times\:{\mathrm{P}}_{3}$	13.28	0.0054	1
$\:{\mathrm{P}}_{2}^{2}\times\:{\mathrm{P}}_{3}$	6.01	0.0367	$\:{\mathrm{P}}_{1}^{2}\times\:{\mathrm{P}}_{4}$	10.83	0.0094	$\:{\mathrm{P}}_{2}^{2}\times\:{\mathrm{P}}_{3}$	3.79	0.0832	1
$\:{\mathrm{P}}_{2}\times\:{\mathrm{P}}_{3}^{2}$	14	0.0046	$\:{\mathrm{P}}_{2}^{2}\times\:{\mathrm{P}}_{3}$	4.68	0.0587	$\:{\mathrm{P}}_{2}\times\:{\mathrm{P}}_{4}^{2}$	59.15	< 0.0001	1
$\:{\mathrm{P}}_{1}^{2}\times\:{\mathrm{P}}_{2}^{2}$	59.71	< 0.0001	$\:{\mathrm{P}}_{1}^{2}\times\:{\mathrm{P}}_{2}^{2}$	11.5	0.008	$\:{\mathrm{P}}_{2}\times\:{\mathrm{P}}_{3}^{2}$	11.8	0.0074	1
$\:{\mathrm{P}}_{1}^{2}\times\:{\mathrm{P}}_{3}^{2}$	10.46	0.0102	$\:{\mathrm{P}}_{1}^{2}\times\:{\mathrm{P}}_{3}^{2}$	7.48	0.023	$\:{\mathrm{P}}_{1}^{2}\times\:{\mathrm{P}}_{3}^{2}$	6.19	0.0345	1
Residual 9									
Cor Total 23									
R^2^ =0.9453, Adj. R^2^ = 0.8602;			R^2^ =0.9561, Adj. R^2^ = 0.8879;			R^2^ = 0.9549, Adj. R^2^= 0.8847;			

**Fig. 7:**
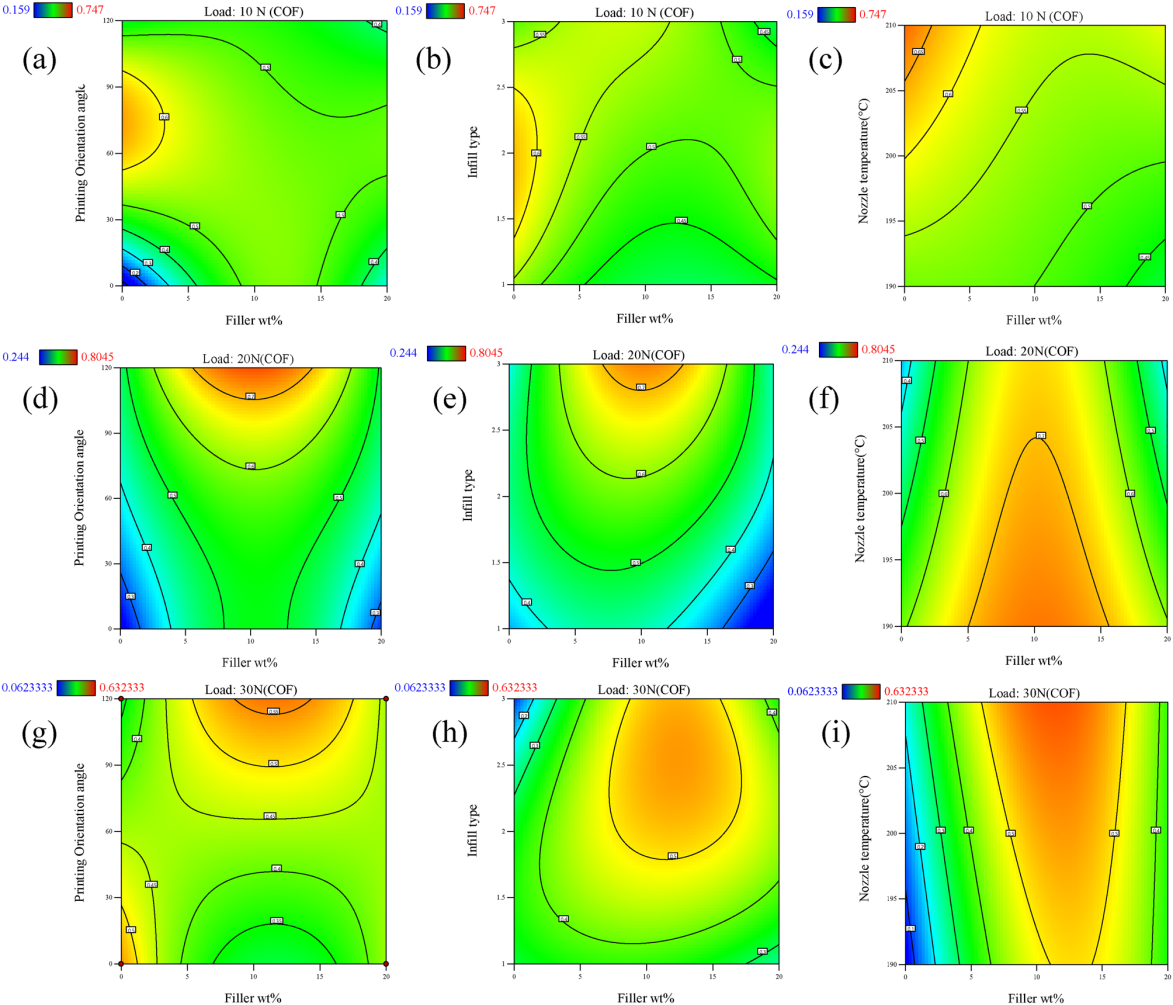
Response surface contour plots showing parameter interactions for COF at 10 N: (**a**) filler wt%–infill type, (b) filler wt%–printing angle, (**c**) printing angle–nozzle temperature; at 20 N: (**d**) filler wt%–infill type, (**e**) filler wt%–printing angle, (**f**) printing angle–nozzle temperature; and at 30 N: (**g**) filler wt%–infill type, (**h**) filler wt%–printing angle, (**i**) printing angle–nozzle temperature.

Figure 7(a-i) illustrates the combined influence of printing parameters, including filler wt%, on the coefficient of friction (COF) of RHBC/PLA composites under different normal loads. At a load of 10 N, Fig. 7(a–c) shows the contour distributions are relatively uniform and dominated by low COF values, indicating stable frictional behavior with weak interaction effects among filler wt%, infill type, printing angle, and nozzle temperature. Similarly, for 20 N, Fig. 7(d–f) presents the contour maps shift toward moderate COF regions with broader gradients, revealing stronger parameter interactions and a noticeable dependence of friction on combined processing conditions. At the highest load of 30 N, Fig. 7(g–i) demonstrates the contours become steeper and more irregular, signifying high sensitivity of COF to small variations in filler content and printing parameters, with localized regions of increased friction. Overall, Fig. 7(a–i) highlights a clear load-dependent evolution of COF, while also demonstrating that suitable combinations of filler wt% and printing conditions can moderate frictional response. This behavior due to a progressive increase in contact stress and real contact area with load, which enhances interfacial resistance, frictional heating, and surface deformation, thereby amplifying the influence of processing parameters on frictional performance at higher normal loads.

### Machine learning prediction

#### Wear rate

**Fig. 8:**
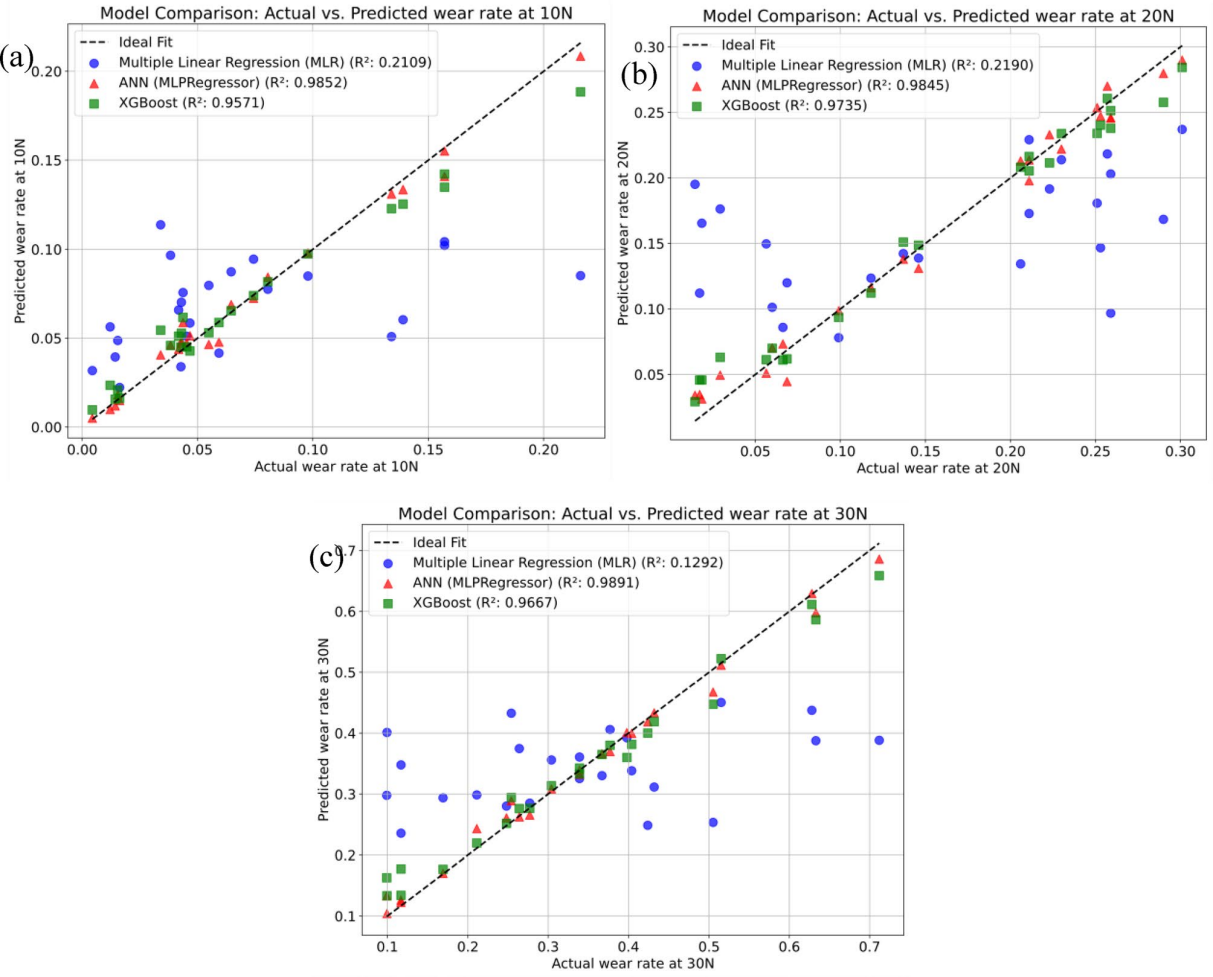
Comparison of experimental and predicted wear rate under varying conditions: (**a**) 10 N, (**b**) 20 N, and (**c**) 30 N for MLR, XGBoost, and ANN models.

Figure 8(a–c) compares the experimentally measured and model-predicted wear rates at loads of 10 N, 20 N, and 30 N using three machine learning models i.e. Multiple Linear Regression (MLR), ANN (MLP Regressor), and XGBoost models. In Fig. 8(a) at 10 N, the ANN predictions closely align with the ideal fit line, achieving a high coefficient of determination (R² = 0.9852), with the lowest error values (RMSE = 0.0065, and MAE = 0.0050), followed by XGBoost (R² = 0.9571, RMSE = 0.0111, MAE = 0.0079), while the MLR model exhibits large scatter with a low R² value of 0.2109, along with higher error values (RMSE = 0.0478, MAE = 0.0369), indicating poor predictive capability. Similarly, Fig. 8(b) at 20 N shows that ANN ((R² = 0.9845, RMSE = 0.0120, MAE = 0.0102) and XGBoost (R² = 0.9735, RMSE = 0.0156, MAE = 0.0124) accurately capture the wear trend with minimal deviation from the ideal fit, whereas MLR again fails to represent the experimental behavior (R² = 0.2190, RMSE = 0.0849, MAE = 0.0670). At the highest load of 30 N, Fig. 8(c), the nonlinear nature of wear becomes more pronounced; ANN demonstrates excellent agreement with experimental data (R² = 0.9891, RMSE = 0.0175, MAE = 0.0119) followed by XGBoost (R² = 0.9667, RMSE = 0.0306, MAE = 0.0230), while MLR shows significant dispersion and the lowest accuracy (R² = 0.1292, RMSE = 0.1568, MAE = 0.1245). Figure 8(a–c) clearly confirms that wear rate is governed by strong nonlinear interactions between process parameters and applied load, which are effectively captured by ANN and XGBoost models, whereas linear regression is inadequate for reliable wear prediction under varying tribological conditions.

**Fig. 9:**
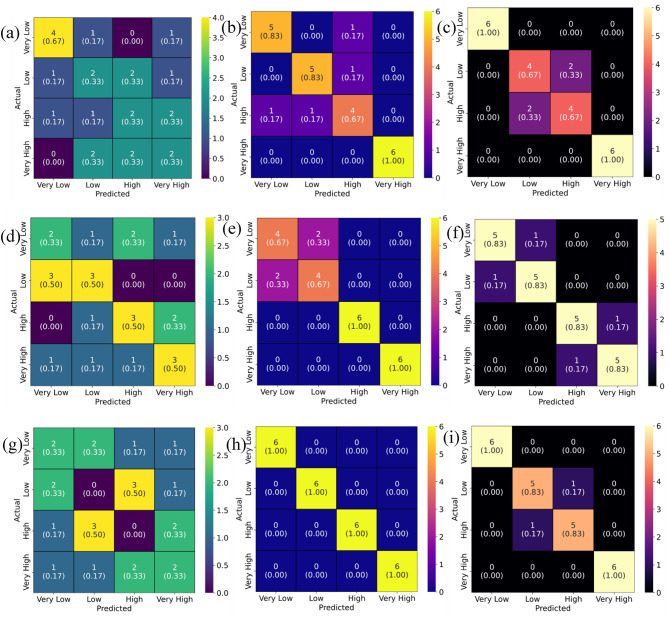
Confusion matrices for wear rate classification of RHBC/PLA composites: (**a–c**) MLR, XGBoost, and ANN at 10 N; (**d–f**) MLR, XGBoost, and ANN at 20 N; (**g–i**) MLR, XGBoost, and ANN at 30 N.

Figure 9(a-i) demonstrates a detailed comparison of the wear rate of RHBC/PLA composites at varying normal loads of 10 N, 20 N, and 30 N using different machine learning models, through confusion matrices. The wear rate is categorised as Very Low, Low, High, and Very High. The diagonal values of the matrix indicate correct predictions, while the off-diagonal values indicate misclassification. At 10 N, MLR correctly classifies 10 out of 24 samples (41.7%), whereas both XGBoost and ANN correctly predict 20 out of 24 samples (83.3%), demonstrating strong discrimination among the four wear rate stages as shown in Fig. 9(a-c). Similarly, MLR achieves 11 out of 24 (45.8%), while XGBoost and ANN again classify 20 out of 24 samples correctly (83.3%), indicating consistent performance at the intermediate load of 20 N in Fig. 9(d-f). Under higher loading, in Fig. 9(g-i), MLR predicts 4 out of 24 (16.7%), showing significant confusion between stages; ANN improves further with 22 out of 24 (91.7%), while XGBoost achieves perfect classification with 24 out of 24 correct predictions (100%). These results indicate that XGBoost attains the highest overall accuracy at the highest load, whereas ANN maintains consistently high and stable classification performance across all wear rate stages and loading conditions.

**Fig. 10:**
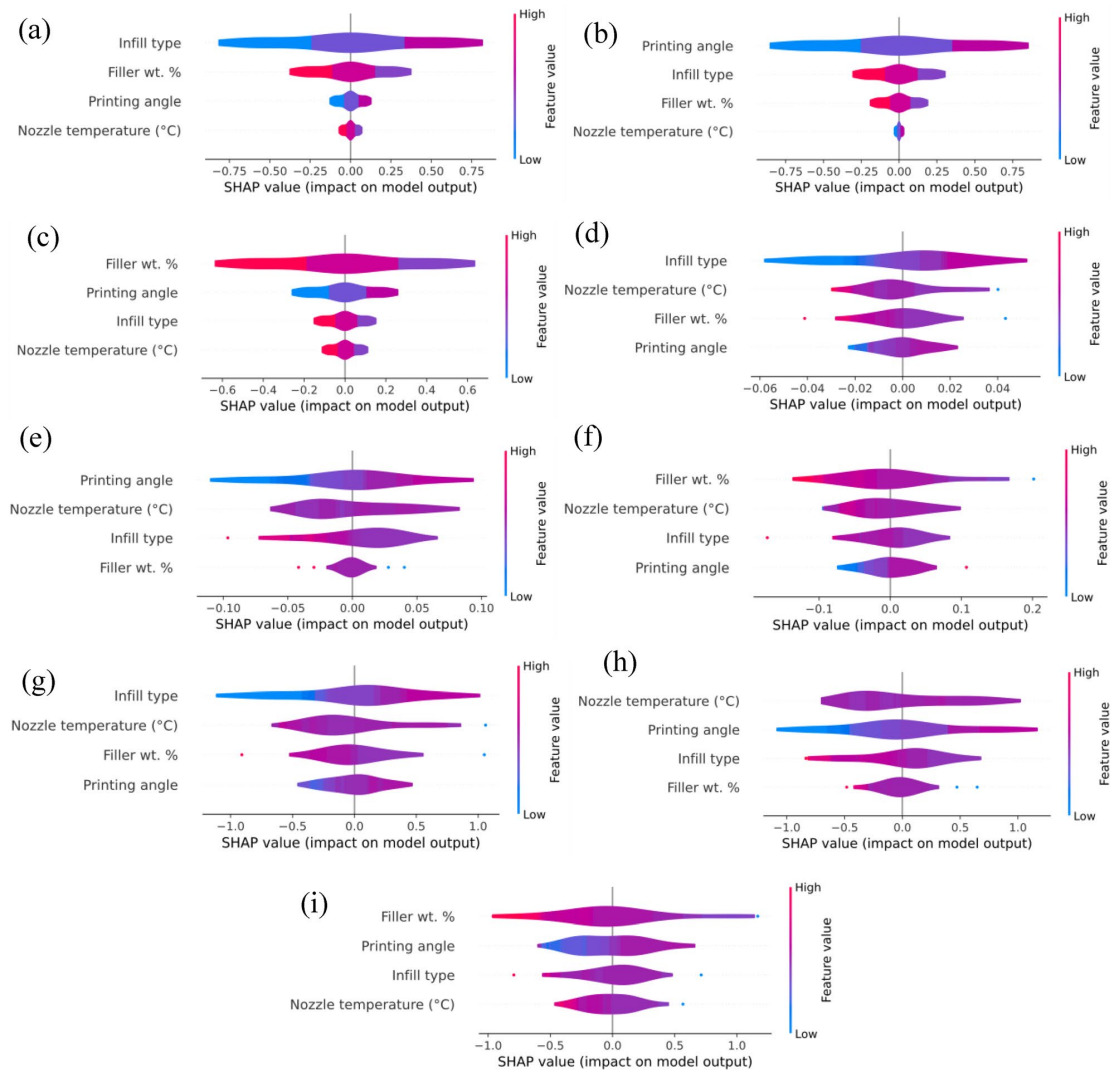
SHAP plots showing feature contributions to wear rate prediction: (**a–c**) MLR at 10, 20, and 30 N; (**d–f**) XGBoost at 10, 20, and 30 N; (**g–i**) ANN at 10, 20, and 30 N.

Figure 10(a-i) illustrates the SHAP plots to investigate the influence of printing parameters, nozzle temperature (°C), printing angle, infill type, including filler wt%, on the predicted wear rate of RHBC/PLA composites under normal loads of 10 N, 20 N, and 30 N. The horizontal axis represents SHAP values, where negative values indicate a reduction and positive values indicate an increase in the predicted wear rate, while the color gradient from blue to pink denotes low to high feature magnitudes. For the MLR model in Fig. 10(a–c), the SHAP values are relatively confined, approximately within − 0.75 to + 0.75 (with plot (c) slightly narrower at about − 0.60 to + 0.60), suggesting moderate and predominantly linear feature influence. In contrast, the XGBoost model in Fig. 10(d–f) exhibits comparatively smaller numerical ranges around − 0.08 to + 0.04 in Fig. 10(d), − 0.10 to + 0.10 in Fig. 10(e), and extending to approximately − 0.10 to + 0.20 in Fig. 10(f) shows clearer separation among features, indicating nonlinear sensitivity captured within a compact prediction scale. The ANN model in Fig. 10(g–i) demonstrates the broadest SHAP distributions, approximately between − 1.0 and + 1.0 across all loads, with infill type and printing angle displaying more pronounced effects at higher loads. Overall, the wider and more distinct SHAP distributions observed in ANN, and to a lesser extent in XGBoost, compared with MLR, indicate their enhanced ability to capture complex nonlinear relationships governing wear behavior, whereas the linear nature of MLR limits its sensitivity to parameter interactions.

**Fig. 11:**
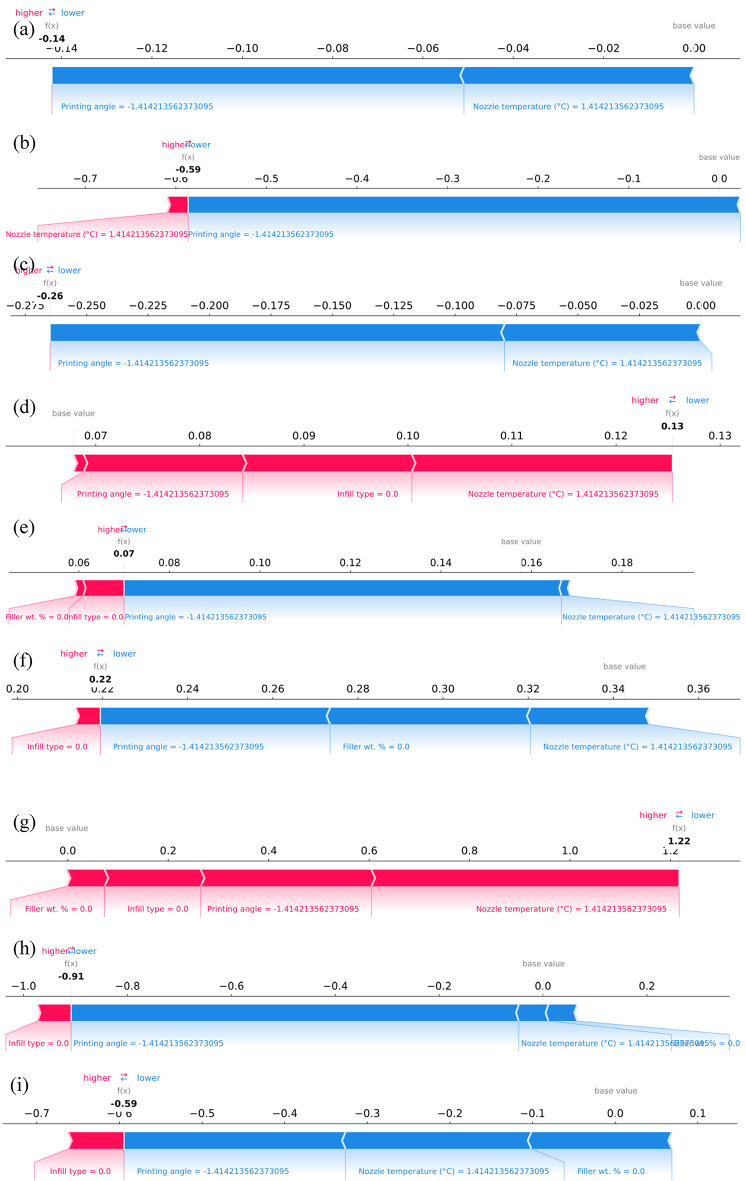
SHAP force plots illustrating parameter contributions to wear rate prediction: (**a–c**) MLR at 10, 20, and 30 N; (**d–f**) XGBoost at 10, 20, and 30 N; (**g–i**) ANN at 10, 20, and 30 N.

Figure 11 illustrates SHAP force plots that quantify the individual and combined effects of printing parameters such as nozzle temperature (°C), printing angle, infill type, and filler wt% on the predicted wear rate at varying normal loads of 10 N, 20 N, and 30 N. The prediction starts from a base value and is shifted left and right, with blue and red colors indicates decrease and increase in wear rate, respectively, depending on the magnitude and direction of feature contributions. For MLR, the deviations are relatively minimal and additive: in Fig. 11(a), the prediction shifts from ~ 0.00 to about − 0.05, in Fig. 11(b) to nearly − 0.70, and in Fig. 11(c) to around − 0.25, mainly due to the combined negative influence of printing angle and nozzle temperature. In XGBoost, stronger nonlinear behavior is evident, with the prediction increasing from ~ 0.07 to ~ 0.13 in Fig. 11(d), and decreasing from ~ 0.16 to ~ 0.07 in Fig. 11(e) and from ~ 0.34 to ~ 0.22 in Fig. 11(f), largely driven by infill type and printing angle. ANN demonstrates the largest deviations, with the output rising to ~ 1.22 in Fig. 11(g) and decreasing to approximately − 0.91 and − 0.59 in Fig. 11(h) and Fig. 11(i), respectively. These larger shifts confirm that ANN, followed by XGBoost, more effectively captures complex nonlinear interactions governing wear behavior compared to the linear MLR model.

**Fig. 12:**
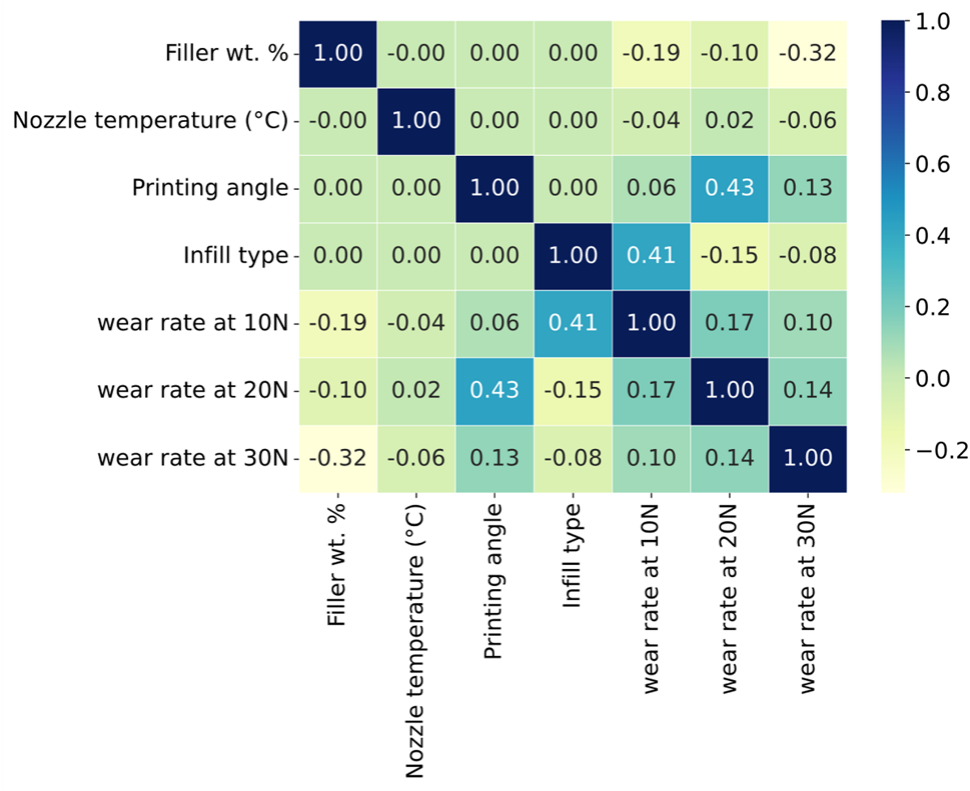
Correlation heat map for wear rate at different normal loads.

The correlation matrix for wear rate reveals distinct load-dependent relationships between processing parameters and tribological response in Fig. 12. At 10 N, wear rate shows a moderate positive correlation with infill type (*r* = 0.41), indicating that internal structural configuration significantly affects material removal at lower load. Printing angle exhibits a weak positive correlation (*r* = 0.06), while filler wt% shows a weak negative correlation (*r* = − 0.19), suggesting that increasing filler content slightly reduces wear. Nozzle temperature has a negligible negative influence (*r* = − 0.04). At 20 N, printing angle becomes the dominant parameter (*r* = 0.43), demonstrating stronger sensitivity at intermediate load, whereas infill type shifts to a weak negative relationship (*r* = − 0.15). Filler wt% (*r* = − 0.10) continues to show a minor wear-reducing trend, and nozzle temperature remains insignificant (*r* = 0.02). At 30 N, filler wt% exhibits the strongest negative correlation (*r* = − 0.32), indicating improved wear resistance at higher reinforcement levels under elevated load. Printing angle maintains a weak positive effect (*r* = 0.13), and infill type (*r* = − 0.08) and nozzle temperature (*r* = − 0.06) show minimal influence. Inter-load correlations are modest (*r* = 0.17 between 10 N and 20 N; *r* = 0.14 between 20 N and 30 N), confirming that wear mechanisms vary with applied load.

#### Coefficient of friction (COF)

**Fig. 13:**
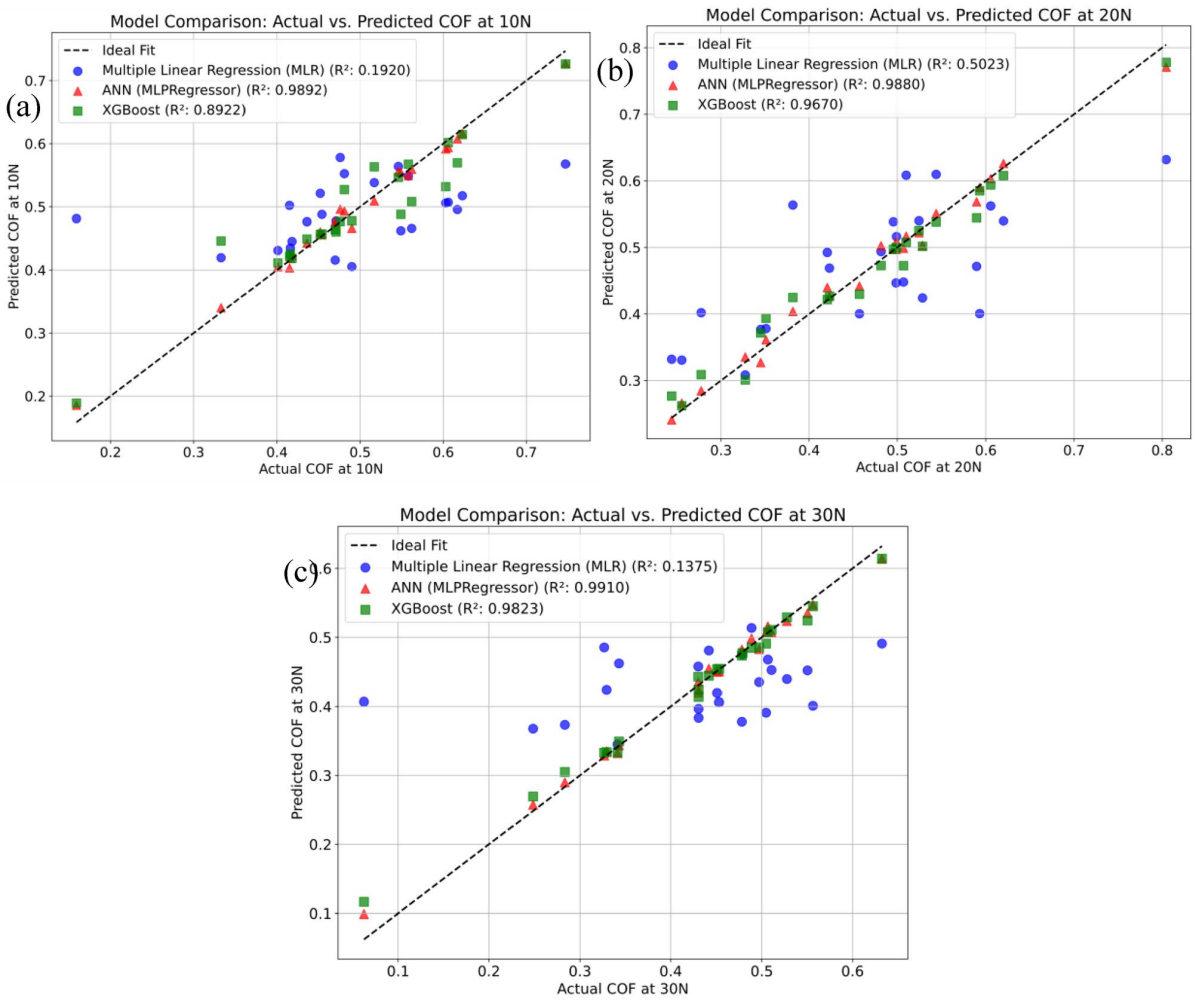
Comparison of experimental and predicted coefficient of friction (COF) under varying conditions: (**a**) 10 N, (**b**) 20 N, and (**c**) 30 N for MLR, XGBoost, and ANN models.

Figure 13(a–c) demonstrates the comparison of the experimentally measured and model-predicted coefficient of friction (COF) for specimens produced with varying printing parameters and filler wt% under normal loads of 10, 20, and 30 N. At 10 N in Fig. 13(a), the ANN model exhibits excellent agreement with the experimental COF values (R² = 0.9892, RMSE = 0.0118, MAE = 0.0098), closely followed by XGBoost (R² = 0.8922, RMSE = 0.0372, MAE = 0.0246), whereas the MLR model shows wide scatter and poor accuracy (R² = 0.1920, RMSE = 0.1019, MAE = 0.0778). At 20 N, Fig. 13(b), demonstrates that the predictive capability of ANN remains high (R² = 0.9880, RMSE = 0.0141, MAE = 0.0112), followed by XGBoost (R² = 0.9670, RMSE = 0.0233, MAE = 0.0178), while MLR improves slightly but still shows limited reliability (R² = 0.5023, RMSE = 0.0907, MAE = 0.0748). Under the highest load of 30 N, in Fig. 13(c), ANN continues to demonstrate superior prediction accuracy (R² = 0.9910, RMSE = 0.0112, MAE = 0.0083), followed by XGBoost (R² = 0.9823, RMSE = 0.0158, MAE = 0.0107), whereas MLR again performs inadequately (R² = 0.1375, RMSE = 0.1103, MAE = 0.0850). The observed trend reflects the strong nonlinear dependence of COF on the combined variation of filler wt% and printing parameters, which becomes more pronounced with increasing normal load due to changes in real contact area, frictional heating, interfacial adhesion, and surface deformation. ANN and XGBoost effectively capture these effects, but linear regression does not.

**Fig. 14:**
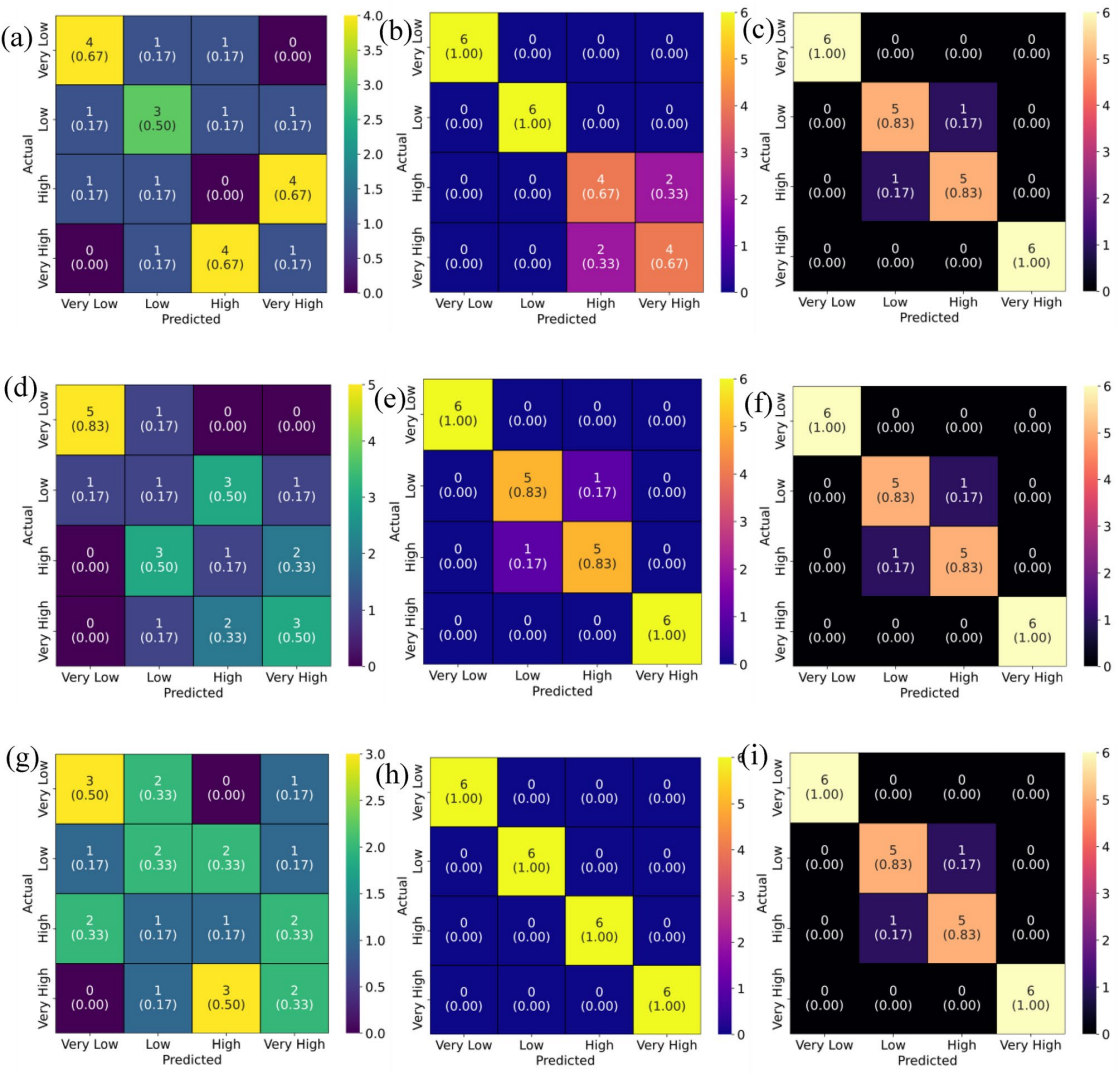
Confusion matrices for COF classification of RHBC/PLA composites: (**a–c**) MLR, XGBoost, and ANN at 10 N; (**d–f**) MLR, XGBoost, and ANN at 20 N; (**g–i**) MLR, XGBoost, and ANN at 30 N.

Figure 14(a-i) presents the confusion matrices for COF (coefficient of friction) classification of RHBC/PLA composites at normal loads of 10 N, 20 N, and 30 N using Multiple Linear Regression (MLR), XGBoost, and Artificial Neural Network (ANN). The COF values are categorized into four stages: Very Low, Low, High, and Very High. In each matrix, the diagonal elements represent correct predictions, while the other elements indicate misclassification between stages. At 10 N, MLR correctly classifies 8 out of 24 samples (33.3%), showing notable confusion among adjacent COF categories in Fig. 14(a). In contrast, both XGBoost and ANN correctly predict 22 out of 24 samples (91.7%), demonstrating strong classification capability in Fig. 14(b-c). At 20 N, MLR improves slightly to 10/24 (41.7%), whereas XGBoost achieves perfect classification with 24/24 (100%), and ANN maintains high performance at 22/24 (91.7%) in Fig. 14(d-f). Figure 14(g-i) presents MLR values of 10/24 (41.7%), reflecting persistent inter-stage misclassification, while XGBoost sustains 100% accuracy, and ANN continues to achieve 22/24 (91.7%) at 30 N. The results indicate that XGBoost attains the highest accuracy at higher loads, ANN maintains consistently strong predictive performance across all loading conditions, and MLR exhibits comparatively lower effectiveness due to greater confusion among COF classification stages.

**Fig. 15:**
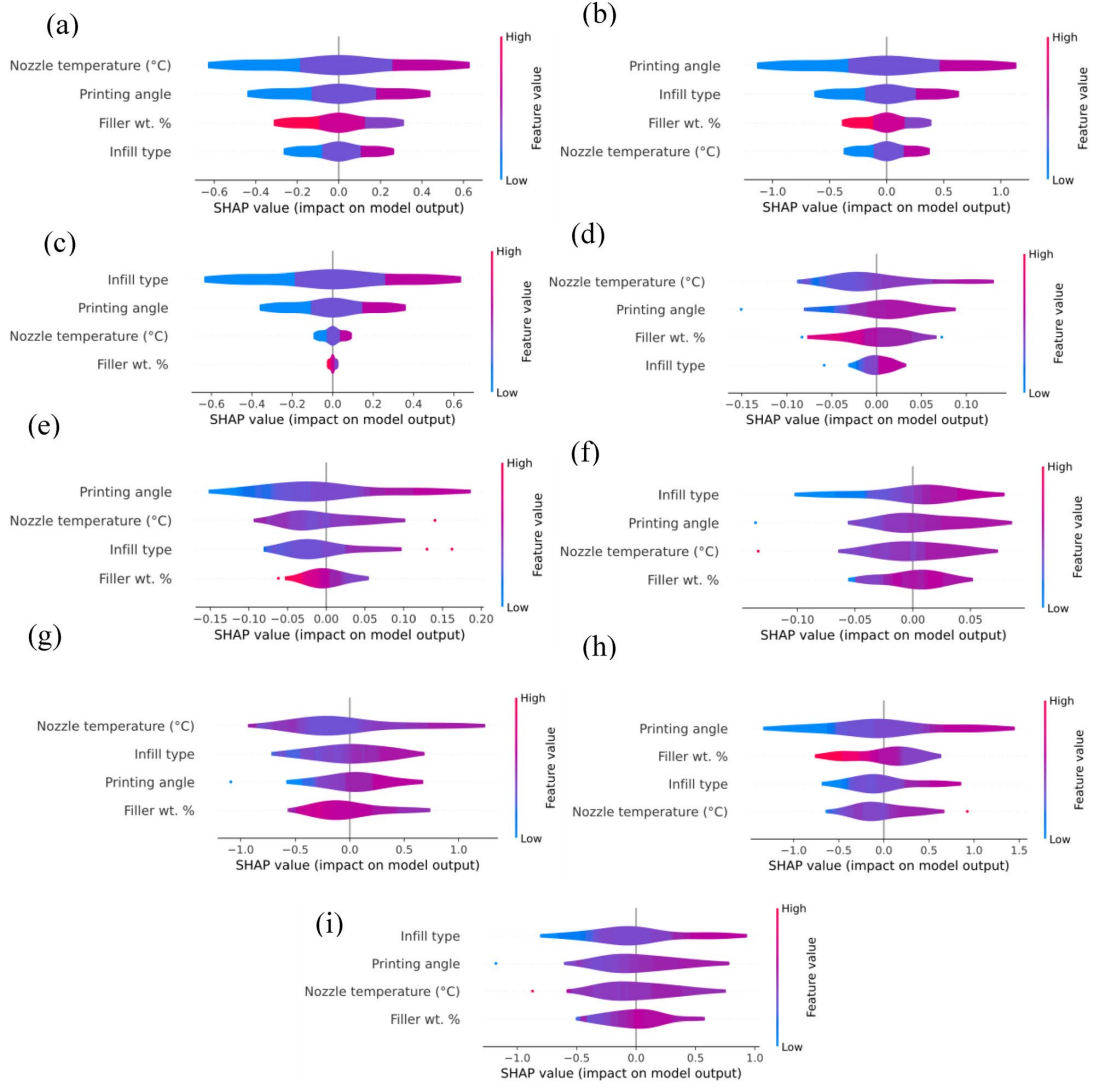
SHAP plots showing feature contributions to COF prediction: (**a–c**) MLR at 10, 20, and 30 N; (**d–f**) XGBoost at 10, 20, and 30 N; (**g–i**) ANN at 10, 20, and 30 N.

**Fig. 16:**
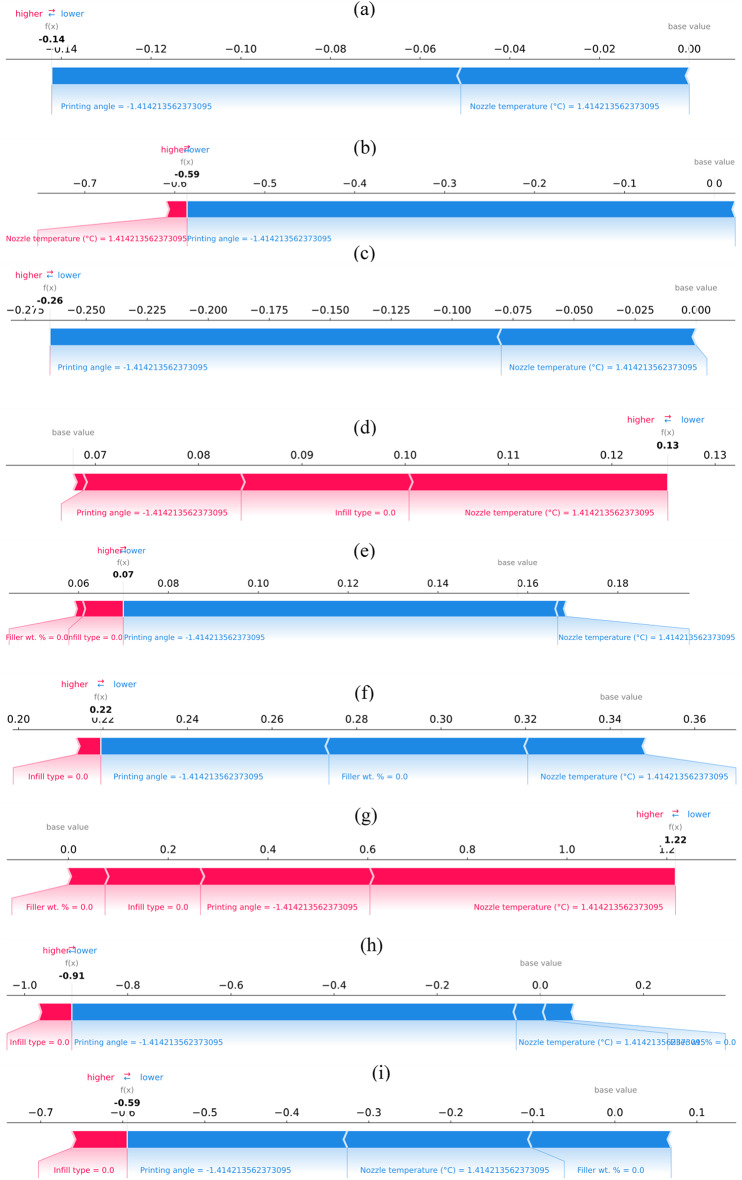
SHAP force plots showing parameter contributions to COF prediction: (**a–c**) MLR at 10, 20, and 30 N; (**d–f**) XGBoost at 10, 20, and 30 N; (**g–i**) ANN at 10, 20, and 30 N.

Figure 15(a-i) illustrates the contribution of printing parameters, including filler wt% by SHAP plots, to the predicted COF of RHBC/PLA composites at varying normal loads of 10 N, 20 N, and 30 N. The horizontal axis represents SHAP values, i.e., impact on model output, with negative values reducing and positive values increasing the predicted COF, while the color gradient from blue to pink denotes low to high feature values. Figure 15(a-c) presents relatively narrow and generally within approximately − 0.6 to 0.6 SHAP values, indicating limited feature influence; nozzle temperature and printing angle exhibit slightly wider spreads compared to infill type and filler wt%. Similarly, Fig. 15(d-f) illustrates that SHAP values range from − 0.15 to + 0.15, which is considerable in certain cases, particularly for printing angle and nozzle temperature, highlighting stronger nonlinear contributions. ANN result is presented in Fig. 16(g–i), which also shows broad SHAP distributions, typically between − 1.0 and + 1.0, with infill type and printing angle displaying prominent impact at higher loads. The broader and more distinct SHAP distributions observed in XGBoost and ANN compared to MLR justify their superior predictive capability, as these models effectively capture complex nonlinear interactions influencing COF, whereas MLR is constrained by linear assumptions, resulting in reduced sensitivity to parameter variations.

Figure 16(a-i) demonstrates that, the significant contribution of printing parameters such as nozzle temperature (°C), printing angle, and infill type, including filler wt% to the predicted coefficient of friction (COF) at varying normal loads of 10 N, 20 N, and 30 N for different machine learning models such as MLR in Fig. 16(a–c), XGBoost in Fig. 16(d–f), and ANN in Fig. 16(g–i). Each prediction starts from a base value and shifts to the left side, with blue color, indicating a reduction in COF, or right side with red color, indicating an increase, depending on the magnitude of feature influence. For MLR in Fig. 16(a–c), the deviations from the base value are relatively moderate and additive: in Fig. 16(a) the prediction shifts from ~ 0.00 to approximately − 0.05, in Fig. 16(b) to nearly − 0.59, and in Fig. 16(c) to about − 0.25, primarily due to the combined negative contributions of printing angle and nozzle temperature. Similarly, for XGBoost in Fig. 16(d–f), more distinct nonlinear effects are observed, with the prediction increasing from ~ 0.07 to ~ 0.13 in Fig. 16(d), while decreasing from ~ 0.16 to ~ 0.07 in Fig. 16(e) and from ~ 0.34 to ~ 0.22 in Fig. 16(f), largely influenced by infill type and printing angle. Finally, for ANN in Fig. 16(g–i) exhibits the most pronounced shifts, with the COF rising from ~ 0.00 to ~ 1.22 in Fig. 16(g) and decreasing to approximately − 0.91 and − 0.59 in Fig. 16(h) and Fig. 16(i), respectively. These larger deviations confirm that ANN, followed by XGBoost, captures stronger nonlinear interactions affecting COF, whereas MLR remains limited by its linear structure.

**Fig. 17:**
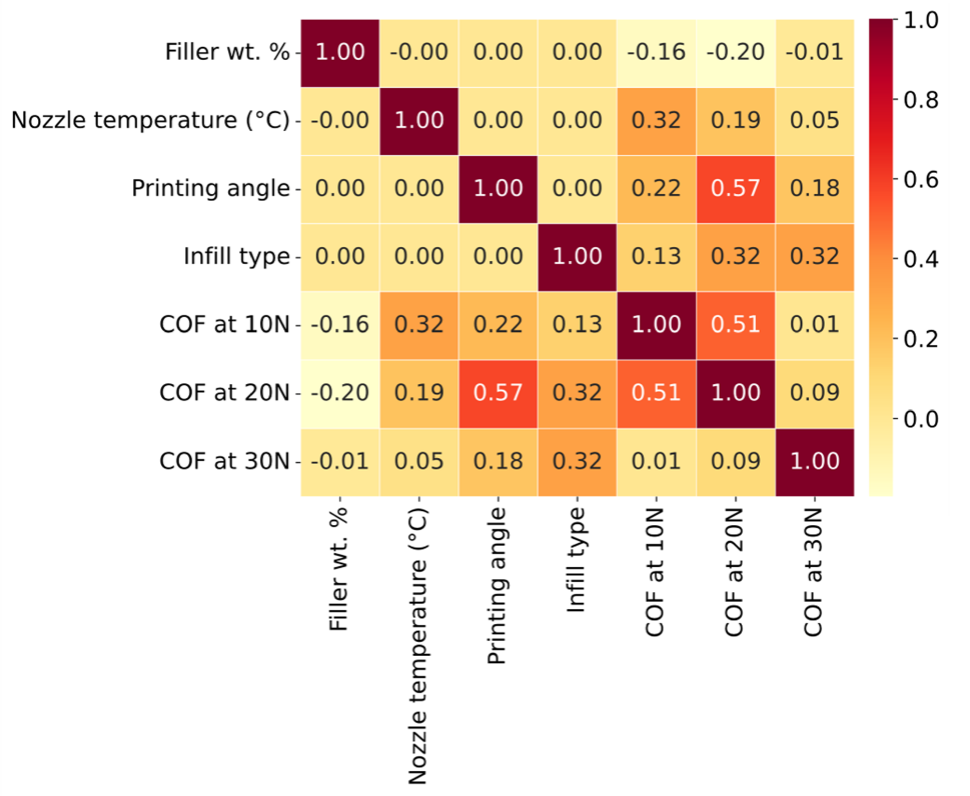
Correlation heat map for COF at different normal loads.

Figure 17 demonstrates correlation matrix graph for COF, which demonstrates a clear load-dependent behavior with varying strengths of association for each processing parameter. At 10 N, COF shows moderate positive correlation with nozzle temperature (*r* = 0.32), indicating that higher extrusion temperatures tend to increase friction at lower load. Printing angle exhibits a weaker positive correlation (*r* = 0.22), suggesting a noticeable but secondary influence of raster orientation. Infill type presents a mild positive relationship (*r* = 0.13), whereas filler wt% shows a weak negative correlation (*r* = − 0.16), implying that increasing reinforcement slightly reduces friction. At 20 N, printing angle becomes the dominant parameter with a strong positive correlation (*r* = 0.57), confirming significant frictional sensitivity to structural orientation at intermediate load. Infill type also shows a moderate positive effect (*r* = 0.32), while nozzle temperature has a weaker positive association (*r* = 0.19). Filler wt% maintains a negative trend (*r* = − 0.20), indicating friction reduction with higher filler content. At 30 N, infill type remains moderately correlated (*r* = 0.32), whereas printing angle (*r* = 0.18) and nozzle temperature (*r* = 0.05) exert relatively weak effects; filler wt% becomes nearly neutral (*r* = − 0.01). Inter-load correlations show a strong relationship between COF at 10 N and 20 N (*r* = 0.51), while correlations involving 30 N are weak (*r* = 0.09 and *r* = 0.01), suggesting altered frictional mechanisms at higher load.

## Conclusion

This present study focused on the optimisation of the tribological behavior of RHBC/PLA composite by varying the printing parameters, and filler wt%, by statistical analysis and machine learning (ML) models (i.e. Multiple Linear Regression (MLR), Extreme Gradient Boosting (XGBoost), and Artificial Neural Networks (ANN)). The key findings are as follows:

- RHBC/PLA composites exhibited improved tribological performance, with reduced wear rate and COF under optimized conditions.
- With increasing load, wear rate increased, showing a transition from mild to severe wear; PLA exhibited dominant ploughing, while RHBC/PLA showed particle pull-out, pitting, and third-body effects.
- ANOVA identified load as the dominant factor for wear rate (R² > 0.91), while COF is governed by nonlinear interactions of process parameters (F up to 67.1, *p* < 0.05).
- ANN achieved the highest prediction accuracy (R² ≈ 0.99) for both wear rate and COF, outperforming XGBoost and MLR.
- SHAP force analysis showed that nozzle temperature, printing angle, infill type, and filler wt% significantly influence COF, with ANN capturing stronger nonlinear effects.
- Correlation analysis revealed load-dependent behaviour: nozzle temperature dominates at 10 N (*r* ≈ 0.32), printing angle at 20 N (*r* ≈ 0.57), and infill type at 30 N (*r* ≈ 0.32), while filler wt% generally reduces COF.

The present study is limited by the relatively small dataset derived from a structured experimental design and the restricted range of filler content (10–20 wt%), which constrains the generalization of the developed models beyond the investigated domain. Future work should therefore focus on generating larger datasets with a wider range of filler compositions to improve model robustness and applicability. Additionally, further studies may consider multi-response optimization of RHBC/PLA composites by integrating mechanical, and thermal responses^42–44^. Future work should focus on expanding the experimental design by incorporating additional FDM parameters and adopting advanced optimization techniques. It should also extend to natural fiber–reinforced and metal matrix composites for broader applications such as bearings, bushings, structural, and sliding components^45,46^.

## Data Availability

Data is provided within the manuscript.

## References

[1] Kapoor, D. D., Madaan, P., Kumar, J., Singh, R. & Tyagi, Y. K. Transition towards renewable and biodegradable polymers: a comprehensive review. *J. Polym. Res.* **32**, 345. 10.1007/s10965-025-04524-8 (2025).

[2] Dallaev, R., Papež, N., Allaham, M. M. & Holcman, V. Biodegradable Polymers: Properties, Applications, and Environmental Impact. Polymers, 17(14), 1981. (2025). 10.3390/polym17141981 10.3390/polym17141981 PMC12298952 40732859

[3] Beena Unni, A. & Muringayil Joseph, T. Enhancing Polymer Sustainability: Eco-Conscious Strategies. *Polymers* **16** (13), 1769. 10.3390/polym16131769 (2024). 39000625 10.3390/polym16131769 PMC11244229

[4] Soni, A. et al. An overview of recent trends and future prospects of sustainable natural fiber-reinforced polymeric composites for tribological applications. *Ind. Crops Prod.* **222**, 119501. 10.1016/j.indcrop.2024.119501 (2024).

[5] Olonisakin, K., Mohanty, A. K., Thimmanagari, M. & Misra, M. Recent advances in biodegradable polymer blends and their biocomposites: a comprehensive review. *Green Chem.* **27** (38), 11656–11704. 10.1039/D5GC01294E (2025).

[6] Guo, Y., Wang, X., Zhang, Z., Zhang, X. & Li, Y. Recent advances in nanofillers for polymer tribological enhancement: From applications and classification to mechanisms. *Mater. Today Commun.* **49**, 113886. 10.1016/j.mtcomm.2025.113886 (2025).

[7] Laraba, S. R. et al. Enhancing the tribological performance of PLA-based biocomposites reinforced with graphene oxide. *J. Mech. Behav. Biomed. Mater.* **148**, 106224. 10.1016/j.jmbbm.2023.106224 (2023). 37944226 10.1016/j.jmbbm.2023.106224

[8] Al Abir, A., Chakrabarti, D. & Trindade, B. Fused Filament Fabricated Poly(lactic acid) Parts Reinforced with Short Carbon Fiber and Graphene Nanoparticles with Improved Tribological Properties. *Polymers* **15** (11), 2451. 10.3390/polym15112451 (2023). 37299249 10.3390/polym15112451 PMC10255553

[9] Upadhyay, R. K., Mishra, A. K. & Kumar, A. Mechanical degradation of 3D printed PLA in simulated marine environment. *Surf. Interfaces*. **21**, 100778. 10.1016/j.surfin.2020.100778 (2020).

[10] Fouly, A., Assaifan, A. K., Alnaser, I. A., Hussein, O. A. & Abdo, H. S. Evaluating the Mechanical and Tribological Properties of 3D Printed Polylactic-Acid (PLA) Green-Composite for Artificial Implant: Hip Joint Case Study. *Polymers* **14** (23), 5299. 10.3390/polym14235299 (2022). 36501692 10.3390/polym14235299 PMC9738854

[11] Hanon, M. M., Alshammas, Y. & Zsidai, L. Effect of print orientation and bronze existence on tribological and mechanical properties of 3D-printed bronze/PLA composite. *Int. J. Adv. Manuf. Technol.* **108**, 553–570. 10.1007/s00170-020-05391-x (2020).

[12] Zawadzki, P. et al. Tribological properties of fused deposition modeling-printed polylactic acid and PLA-CF: extrusion temperature and internal structure. *J. Tribol.* **148** (2), 024202. 10.1115/1.4068991 (2026).

[13] Santo, J., Pradhik, V., Kalakoti, S., Saravanan, P. & Penumakala, P. K. Effect of composite processing technique on tribological properties of 3D printed PLA-graphene composites. *Tribol. Int.* **198**, 109895. 10.1016/j.triboint.2024.109895 (2024).

[14] Keshavamurthy, R. et al. Mechanical and Wear Studies of Boron Nitride-Reinforced Polymer Composites Developed via 3D Printing Technology. *Polymers* **15** (22), 4368. 10.3390/polym15224368 (2023). 38006092 10.3390/polym15224368 PMC10675459

[15] Albahkali, T., Abdo, H. S., Salah, O. & Fouly, A. Adaptive Neuro-Fuzzy-Based Models for Predicting the Tribological Properties of 3D-Printed PLA Green Composites Used for Biomedical Applications. *Polymers* **15** (14), 3053. 10.3390/polym15143053 (2023). 37514443 10.3390/polym15143053 PMC10383854

[16] Lasch, G. et al. Exploring the Tribological Properties of 3D Printed Polymer Composites: An Experimental Study. *Polym. Compos.* **46** (1), 112–128. 10.1002/pc.70591 (2025).

[17] Arunadevi, M., Bhandarkar, V. N. V. & Keshavamurthy, R. Prediction of Mechanical Properties of Additively Manufactured Parts Using Machine Learning Techniques. *J. Inst. Eng. India Ser. D*. 10.1007/s40033-025-00905-x (2025).

[18] Raj, T. M., Mahendran, G., Sarma, D. & Sivaperumal, M. Characterization study on mechanical, flammability and wear behavior of 3D-printed PLA composites reinforced with surface treated citrus maxima fruit peel biocarbon. *Polym. Bull.* **82** (15), 9805–9819. 10.1007/s00289-024-05561-w (2025).

[19] Ju, G. et al. Mechanical and Tribological Properties of Rice Husk Biochar-Reinforced Glass Fiber/Ultra‐High Molecular Weight Polyethylene Composites for Water‐Lubricated Bearings. *Polym. Compos.* **47** (4), 3618–3637. 10.1002/pc.29263 (2026).

[20] Louhichi, B. et al. Bibliometric analysis, current studies, and future perspective of biochar filled polymer composite: a sustainable filler for enhancing physical properties. *Adv. Physics: X*. **11** (1), 2646244. 10.1080/23746149.2026.2646244 (2026).

[21] Rabbaje, F., Nimbona, F., Taleb, A. A., El-Hami, A. & El-Hami, K. Parametric simulation based FDM printed PLA gears and process parameters effects on mechanical performance. *Int. J. Adv. Manuf. Technol.* **130**, 1145–1160. 10.1007/s00170-026-17671-z (2026).

[22] Sundarasetty, H. et al. Machine learning guided process optimization and sustainable valorization of coconut biochar-filled PLA biocomposites. *Sci. Rep.* **15**, 34647. 10.1038/s41598-025-10492-w (2025). 41047416 10.1038/s41598-025-19791-0 PMC12497883

[23] Boukhatem, G., Bencheikh, M., Bekkouche, S. R., Benhabib, A. & Gherrabi, A. Data-driven optimization and pressuremeter modulus prediction using response surface methodology for smarter geotechnical design. *Sci. Rep.* **16**, 5679. 10.1038/s41598-026-36262-2 (2026). 41554812 10.1038/s41598-026-36262-2 PMC12891667

[24] Abumelha, H. M. et al. Evaluation of tetracycline removal by magnetic metal organic framework from aqueous solutions: Adsorption isotherm, kinetics, thermodynamics, and Box-Behnken design optimization. *J. Saudi Chem. Soc.* **27**, 101706. 10.1016/j.jscs.2023.101706 (2023).

[25] Harishbabu, S., Alrasheedi, N. H., Louhichi, B., Sahu, S. K. & Ma, Q. Optimization and Prediction of Mechanical Properties of Additively Manufactured PLA/GNP Composites via Response Surface Methodology and Machine Learning Models. *Polymers* **17** (21), 2894. 10.3390/polym17212894 (2025). 41228655 10.3390/polym17212894 PMC12608263

[26] Kohan-Baghkheirati, E. et al. Hybrid Response Surface Methodology-Support Vector Machine modeling approach enhanced the efficiency of optimization for sansevieria propagation. *Planr Cell Tissue Organ Cult.* **164**, 10. 10.1007/s11240-025-03323-9 (2026).

[27] Harishbabu, S., Alrasheedi, N. H., Louhichi, B., Sreekanth, P. S. R. & Sahu, S. K. Machine Learning-Assisted Synergistic Optimization of 3D Printing Parameters for Enhanced Mechanical Properties of PLA/Boron Nitride Nanocomposites. *Machines* **13** (10), 949. 10.3390/machines13100949 (2025).

[28] Uddin, M. S., Sidek, M. F. R., Faizal, M. A., Ghomashchi, R. & Pramanik, A. Evaluating mechanical properties and failure mechanisms of fused deposition modeling acrylonitrile butadiene styrene parts. *J. Manuf. Sci. Eng.* **139** (8), 081018. 10.1115/1.4036713 (2017).

[29] Salguero, J., Iglesias, P., Vazquez-Martinez, J. M., Batista, M. & Sol, D. I. A comparative study of disk wear volume evaluation of Al2024 based on ASTM G99. ASME International Mechanical Engineering Congress and Exposition, **V009**T15A002. (2022). 10.1115/IMECE2022-94625

[30] Sidh, K. N. & Hirani, H. Enhanced wear volume analysis using three-dimensional profilometry for four-ball tribometer setups. *J. Tribol.* **147** (9), 091110. 10.1115/1.4068170 (2025).

[31] Devadiga, U. & Fernandes, P. Taguchi analysis for sliding wear characteristics of carbon nanotube-flyash reinforced aluminium nanocomposites. *Heliyon* **7** (2), e06170. 10.1016/j.heliyon.2021.e06170 (2021). 33644462 10.1016/j.heliyon.2021.e06170 PMC7887386

[32] Elshaer, R. N., El-Fawakhry, M. K., Mattar, T. & Farahat, A. I. Z. Mathematical modeling of wear behavior and Abbott Firestone zones of 0.16 C steel using response surface methodology. *Sci. Rep.* **12** (1), 14472. 10.1038/s41598-022-18637-3 (2022). 36008539 10.1038/s41598-022-18637-3 PMC9411595

[33] Ul-Hamid, A. Synthesis, microstructural characterization and nanoindentation of Zr, Zr-nitride and Zr-carbonitride coatings deposited using magnetron sputtering. *J. Adv. Res.* **29**, 107–119. 10.1016/j.jare.2020.09.006 (2021). 33842009 10.1016/j.jare.2020.11.010 PMC8020350

[34] Pernica, J. et al. Mechanical Properties of Recycled Polymer Materials in Additive Manufacturing. *Manuf. Technol.* **22** (2), 200–203. 10.21062/mft.2022.025 (2022).

[35] Rath, S., Tripathy, A. & Tripathy, A. R. Prediction of new active cases of coronavirus disease (COVID-19) pandemic using multiple linear regression model. *Diabetes Metabolic Syndrome: Clin. Res. Reviews*. **14** (5), 1467–1474. 10.1016/j.dsx.2020.07.045 (2020). 10.1016/j.dsx.2020.07.045 PMC7395225 32771920

[36] Boominathan, E. et al. Integrating explainable machine learning models for interpreting edm performance in inconel 718 with cryogenically treated electrodes. *Int. J. Adv. Manuf. Technol.* **141**, 1595–1617. 10.1007/s00170-025-16827-7 (2025).

[37] Harishbabu, S. et al. Data-Driven AI Approach for Optimizing Processes and Predicting Mechanical Properties of Boron Nitride Nanoplatelet-Reinforced PLA Nanocomposites. *Polymers* **18** (2), 185. 10.3390/polym18020185 (2026). 41599478 10.3390/polym18020185 PMC12845918

[38] Itano, F., De Abreu De Sousa, M. A. & Del-Moral-Hernandez, E. Extending MLP ANN hyper-parameters Optimization by using Genetic Algorithm. 2018 International Joint Conference on Neural Networks (IJCNN), 1–8. (2018). 10.1109/IJCNN.2018.8489520

[39] Wang, J. et al. Progressive collapse behaviors and mechanisms of 3D printed thin-walled composite structures under multi-conditional loading. *Thin-Walled Struct.* **171**, 108810. 10.1016/j.tws.2021.108810 (2022).

[40] Lancaster, J. K. Abrasive wear of polymers. *Wear* **14** (4), 223–239. 10.1016/0043-1648(69)90047-7 (1969).

[41] Dasari, A., Yu, Z. Z. & Mai, Y. W. Fundamental aspects and recent progress on wear/scratch damage in polymer nanocomposites. *Mater. Sci. Engineering: R: Rep.* **63** (2), 31–80. 10.1016/j.mser.2008.10.001 (2009).

[42] Borah, J. & Chandrasekaran, M. Development of ANN model for predicting mechanical properties of 3D printed PEEK polymer using FDM and optimization of process parameters for better mechanical properties. *Phys. Scr.* **99**, 116005. 10.1088/1402-4896/ad6514 (2024).

[43] Borah, J. & Chandrasekaran, M. Application of Machine Learning-Based Approach to Predict and Optimize Mechanical Properties of Additively Manufactured Polyether Ether Ketone Biopolymer Using Fused Deposition Modeling. *J. Mater. Eng. Perform.* **34**, 19233–19249 10.1007/s11665-024-10629-w (2025).

[44] Maurya, V., Borah, J., Chandrasekaran, M. & Kaushik, A. ML-enabled prediction and optimization of tensile strength and surface characteristics in additively manufactured PETG components. *J. Thermoplast Compos. Mater.*10.1177/08927057261439032 (2026).

[45] Giridharan, K., Elumalai, B. & Vinothkumar, M. Investigation of dry sliding wear and mechanical properties of hybrid epoxy composites reinforced with pineapple leaf and roselle fibers. *Sci. Rep.* **15**, 27991. 10.1007/s43939-025-00475-5 (2025). 40744955 10.1038/s41598-025-10431-1 PMC12314131

[46] Prakash, C., Selvakumar, S. & Manikandan, D. Investigation of micro turning process parameters for titanium alloy using response surface methodology. *Discov Mater.* **6**, 10. 10.1038/s41598-025-10431-1 (2026).

